# Novel age-associated DNA methylation changes and epigenetic age acceleration in middle-aged African Americans and whites

**DOI:** 10.1186/s13148-019-0722-1

**Published:** 2019-08-19

**Authors:** Salman M. Tajuddin, Dena G. Hernandez, Brian H. Chen, Nicole Noren Hooten, Nicolle A. Mode, Mike A. Nalls, Andrew B. Singleton, Ngozi Ejiogu, Kumaraswamy Naidu Chitrala, Alan B. Zonderman, Michele K. Evans

**Affiliations:** 1 Laboratory of Epidemiology and Population Science, National Institute on Aging, NIH Biomedical Research Center, 251 Bayview Boulevard Suite 100 Room 4C-222, Baltimore, MD 21224 USA; 2 Laboratory of Neurogenetics, National Institute on Aging, Bethesda, MD USA; 3 Translational Gerontology Branch, National Institute on Aging, Baltimore, MD USA; 4 Data Tecnica International LLC, Glen Echo, MD USA

**Keywords:** DNA methylation, Epigenetics, Epigenetic clock, Biological age, Aging, Health disparities, Race, European ancestry, African Americans, Epigenome-wide association study

## Abstract

**Background:**

African Americans (AAs) experience premature chronic health outcomes and longevity disparities consistent with an accelerated aging phenotype. DNA methylation (DNAm) levels at specific CpG positions are hallmarks of aging evidenced by the presence of age-associated differentially methylated CpG positions (aDMPs) that are the basis for the epigenetic clock for measuring biological age acceleration. Since DNAm has not been widely studied among non-European populations, we examined the association between DNAm and chronological age in AAs and whites, and the association between race, poverty, sex, and epigenetic age acceleration.

**Results:**

We measured genome-wide DNA methylation (866,836 CpGs) using the Illumina MethylationEPIC BeadChip in blood DNA extracted from 487 middle-aged AA (*N* = 244) and white (*N* = 243), men (*N* = 248), and women (*N* = 239). The mean (sd) age was 48.4 (8.8) in AA and 49.0 (8.7) in whites (*p* = 0.48). We identified 4930 significantly associated aDMPs in AAs and 469 in whites. Of these, 75.6% and 53.1% were novel, largely driven by the increased number of measured CpGs in the EPIC array, in AA and whites, respectively. AAs had more age-associated DNAm changes than whites in genes implicated in age-related diseases and cellular pathways involved in growth and development. We assessed three epigenetic age acceleration measures (universal, intrinsic, and extrinsic). AAs had a significantly slower extrinsic aging compared to whites. Furthermore, compared to AA women, both AA and white men had faster aging in the universal age acceleration measure (+ 2.04 and + 1.24 years, respectively, *p* < 0.05).

**Conclusions:**

AAs have more wide-spread methylation changes than whites. Race and sex interact to underlie biological age acceleration suggesting altered DNA methylation patterns may be important in age-associated health disparities.

**Electronic supplementary material:**

The online version of this article (10.1186/s13148-019-0722-1) contains supplementary material, which is available to authorized users.

## Background

Health disparities are marked differences or inequalities in health measures and indicators, such as morbidity and mortality, between two or more population groups. Health disparities disproportionately affect African Americans (AAs), other racial minorities, and the socioeconomically disadvantaged. The disparities for overall longevity as measured by life expectancy are particularly compelling in the USA where the most pronounced life expectancy gap for AA men who experienced a 20.7-year life expectancy gap when compared to Asian women who had the best overall survival [1]. Nearly two decades later, this troubling trend of lower life expectancy still continues to persist [2]. AAs manifest age-related phenotypes and develop chronic diseases such as cardiovascular diseases, diabetes, and cognitive disorders at younger ages than other demographic groups. This suggests that AAs experience significant rates of premature biological aging. A study conducted in participants of the National Health and Nutrition Examination Survey found that AAs age significantly faster than whites and that biological aging was associated with all-cause mortality rates [3].

The causes of health disparities and its premature aging phenotype are multifactorial and include but are not limited to socioeconomic status, psychosocial stress, genetics, poor access to health care, education, and toxic environmental exposures. However, it has never been fully explained how social determinants of health result in the premature aging phenotype, poor health outcomes, and reduced overall survival. The transduction of a social determinant of health may involve genomic and epigenomic processes that are indeterminate at this time. Among the various epigenetic processes, DNA methylation could be one potential mechanism that may mediate this observed disparity given that DNA methylation is influenced by age, lifestyle, environmental, and host factors [4, 5]. There is evidence from targeted methylation studies that DNA methylation changes are associated with socioeconomic status and age-related diseases [6, 7].

DNA methylation regulates gene expression and maintains genome stability. It is a dynamic process that changes over an individual’s lifespan and is influenced by age and environmental and genetic factors [4]. Further, altered patterns of DNA methylation have been considered as one of the hallmarks of aging and lifespan [8]. Identification of age-associated DNA methylation changes among diverse population groups could provide clues on the epigenetic basis of aging and age-related health disparities among population groups. However, AAs and other racial minorities are underrepresented in epigenetic studies of age-related diseases. Previous studies have identified several age-associated differentially methylated CpG positions (aDMPs) located in genes implicated in chronic diseases and aging [9–28]. However, the majority of these studies were limited by their low genome-wide coverage of CpG sites and also were mostly comprised of populations of European ancestry, therefore precluding the study of the role of DNA methylation in the biology of age-related health disparities among minority populations. The recent development of biological age prediction algorithms based on methylation levels of genome-wide selected CpG sites using elastic net regularized regression methods referred to as DNA methylation age (DNAm age) also known as epigenetic age or the “epigenetic clock,” and the demonstration of robust correlations between DNAm age (epigenetic age) and chronological age provides a valuable research tool to study the social determinants of biological age acceleration [24, 25]. Epigenetic age acceleration has been associated with overall and cause-specific mortality, physical and cognitive function decline, and other aging-related diseases; thus, epigenetic age has been suggested to be a marker of biological age [29–33]. Although sociodemographic and lifestyle factors were shown to accelerate epigenetic aging [32, 34, 35], the interplay between these factors is poorly understood. Specifically, whether race, socioeconomic status, and sex interact with each other to influence accelerated epigenetic age is not known.

The objectives of the present study were (1) to identify novel aDMPs among AAs and whites and (2) to assess the association between race, sex, and poverty status and their interaction on epigenetic age acceleration. We conducted the present study in samples drawn from the Healthy Aging in Neighborhoods of Diversity across the Life Span (HANDLS) study [36]. HANDLS is a population-based longitudinal study of community-dwelling urban AAs and whites aged 30–65 years. Using the Illumina Infinium MethylationEPIC BeadChip, we quantified genome-wide DNA methylation levels at single-CpG dinucleotide resolutions in blood DNA collected from AA and white men and women above and below poverty status. We performed epigenome-wide association analysis of chronological age. We also assessed main effects and interactions between sex, race, and poverty status on epigenetic age acceleration measures. We found that chronological age was associated with widespread DNA methylation changes in various CpG positions and that AAs compared to whites had more aDMPs. These aDMPs were enriched for important genetic regulatory regions, cellular pathways involved in growth and development, and age-related chronic disease susceptibility loci identified by genome-wide association analyses. We also found that AA men had a faster aging corroborating the epidemiologic observations that AA men have a shorter life expectancy.

## Results

### MethylationEPIC array methylation data preprocessing and normalization

We measured DNA methylation using the Illumina Infinium MethylationEPIC BeadChip in blood DNA of 487 participants (244 AAs, 243 whites, 248 men, and 239 women) and 12 technical replicates for quality control. Four hundred seventy participants (50.4% AAs, 50.6% men, and 49.8% above poverty status) passed quality control (Table 1). The age range of the total study participants was 30.2–65.2 years with mean age of 48.7 (standard deviation (sd) = 8.7). The mean (sd) age was 48.4 (8.8) in AAs and 49.0 (8.7) in whites (*p* value = 0.48) indicating no difference in age distributions. Because the EPIC array is a new technology, we compared the performance of different methylation data normalization and preprocessing approaches using methylation levels of technical replicates to identify an optimal method for data preprocessing. After excluding probes with detection *p* value ≥ 0.01, cross-hybridizing probes, and probes containing single nucleotide polymorphisms (minor allele frequency cutoff = 0.05) available in the *DMRcate* package [37], we calculated correlation and probe variance of methylation beta values between technical replicates. Additional file 1: Figure S1a and S1b show the relative performance (correlation and probe variance) of the different methylation data normalization and preprocessing methods: Illumina Genome Studio (Illumina), normal-exponential out-of-band (NOOB), stratified quantile normalization (quantile), subset-quantile within array normalization (SWAN), and no normalization (raw). We found that the NOOB method yielded a higher correlation between technical replicates compared to the other methods.

**Table 1:** Demographic characteristics of the HANDLS study participants with complete DNA methylation data

Characteristics		AAs, *N* = 237	Whites, *N* = 233	*P*
Age, years (sd)		48.4 (8.8)	49.0 (8.7)	0.48
Sex	Men	120	118	1.0
	Women	117	115	
Poverty status	Above	120	114	0.78

*AAs* African Americans, *sd* standard deviation

### Identification of age-associated differentially methylated CpG positions in African Americans and whites

**Fig. 1:**
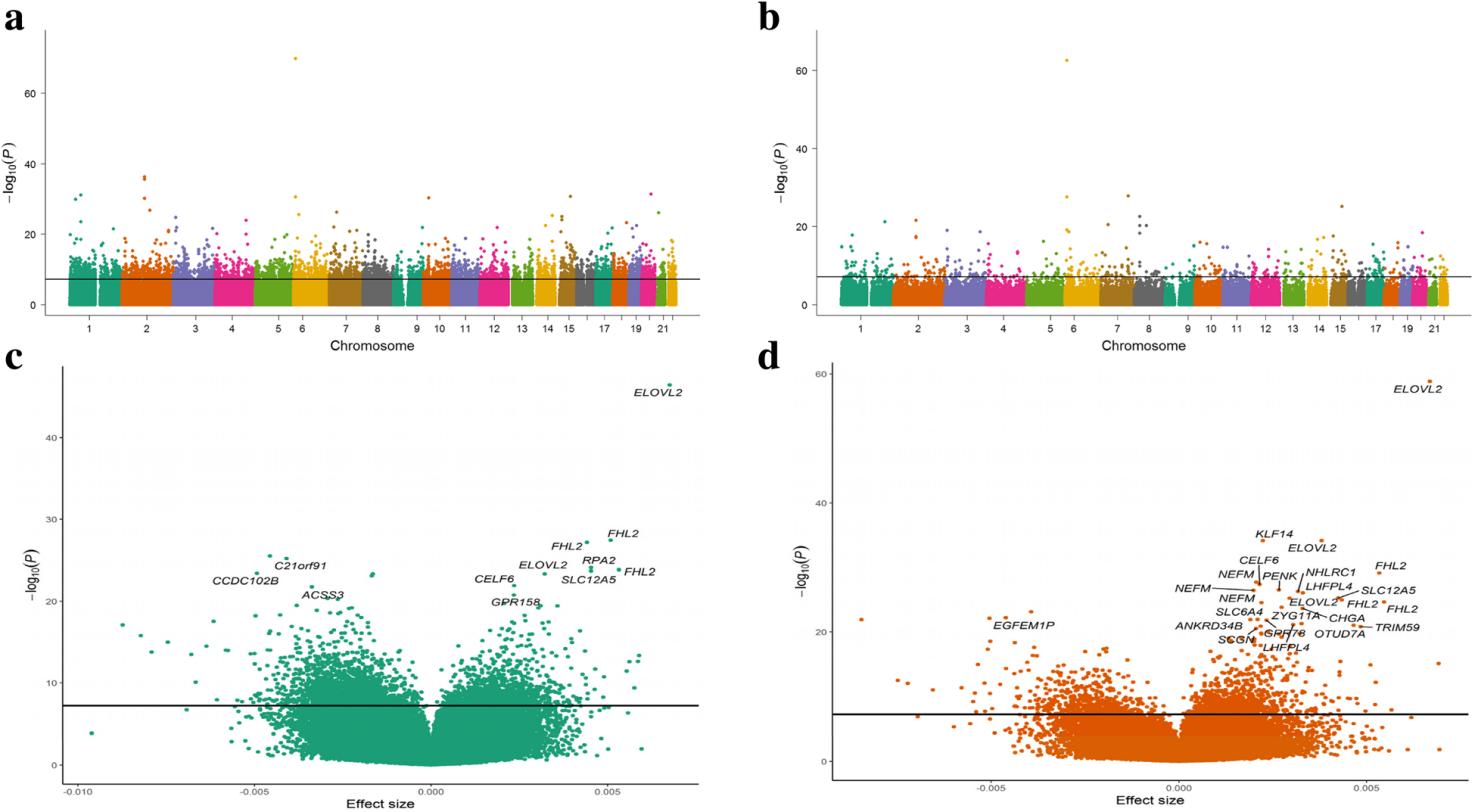
Distribution of age-associated differentially methylated CpG positions (aDMPs) with their effect size in beta values and significance *p* value in the African American (AA) and white participants of the HANDLS study: **a** Manhattan plot in AAs, **b** Manhattan plot in whites, **c** volcano plot in AAs, and **d** volcano plot in whites

**Fig. 2:**
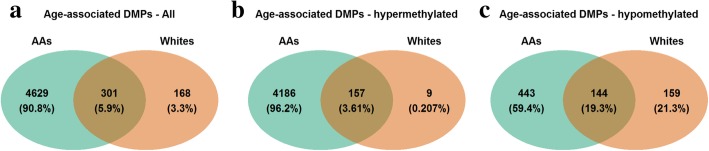
Venn diagrams of significantly age-associated differentially methylated CpG positions (aDMPs) in African Americans (AAs) and whites: **a** overlap of aDMPs between AAs and whites,** b** overlap of aDMPs that were hypermethylated with age, and **c** overlap of aDMPs that were hypomethylated with age

We hypothesized that there would be differences in genome-wide age-associated DNA methylation changes between AAs and whites. To identify age-associated differentially methylated CpG positions (aDMPs), we performed epigenome-wide association analysis of chronological age. We examined the association of baseline chronological age with each of the 765,808 CpG positions that passed quality control separately for AAs (*N* = 237) and whites (*N* = 233). Linear regression models were adjusted for sex, race, poverty status, estimated white blood cell compositions (granulocytes, monocytes, natural killer cells, B cells, CD4^+^, and CD8^+^ T cells), and the first two principal components to account for population stratification. Using a stringent Bonferroni corrected significance threshold and excluding CpGs with effect size between − 0.01 and 0.01, we found significant associations with chronological age for 4930 aDMPs in AAs and 469 aDMPs in whites with effect sizes ranging from − 0.039 to 0.051 in AAs and − 0.050 to 0.047 in whites. Figure 1a and b show the distributions of *p* values of the association between individual CpG positions and chronological age in AAs and whites, respectively. Of these significantly associated aDMPs, 4343 in AAs and 166 in whites gained methylation (hypermethylated) with age (Fig. 1c, d). Although there was substantial overlap of significantly associated aDMPs between AAs and whites, there were more wide-spread age-associated changes (hyper- and hypomethylated) in AAs compared to whites (Fig. 2a, b and c).

Thus far, studies of DNA methylation with chronological age have identified 5321 unique aDMPs in blood-derived DNA using the previous 27K and 450K methylation BeadChips [10, 12–14, 17, 19, 21, 22, 25, 27, 28]. We replicated a number of these previously reported aDMPs in either AAs (1204 aDMPs) or whites (220 aDMPs) with *p* value < 6.53E−08. The list of previously reported aDMPs replicated in our study is shown in Additional file 2: Table S1. Of these previously reported aDMPs, 174 CpG positions including those located in *ELOVL2* replicated in both AAs and whites in the HANDLS study. Previous studies of DNAm and age were conducted in predominantly European ancestry samples and were limited by the number of genome-wide CpG coverage. Using the EPIC array and a balanced sample size of AAs and whites, we found novel aDMPs in both AAs and whites. Of the 4930 aDMPs identified in AAs, 3726 (75.6%) were novel compared with 249 (53.1%) of the 469 aDMPs identified in whites. Table 2 shows the top 50 aDMPs that have not been previously reported. The beta coefficients of these top-ranking aDMPs ranged from − 0.030 to 0.041 in AAs and from − 0.034 to 0.044 in whites. Additional file 3: Figure S2a and b show scatter plots of top ten aDMPs and their corresponding Pearsons’s correlation coefficient with age in AAs and whites, respectively. Some of the top novel age-methylation associations identified in both AAs and whites include CpG positions located in *FGF14*, *FHL2*, *C1QC*, *CELF6*, *NEFM*, and *LHFPL4*. The top unique age-methylation association in AAs were *C21orf91*, *JAZF1*, *NEURL1*, and *ADGRB2*, and in whites were *SLC25A21*, *CPED1*, *NRXN3*, and *OTUD7A* (Table 2).

**Table 2:** Novel age-associated differentially methylated CpG positions (aDMPs) identified using the MethylationEPIC BeadChip

I. African Americans								II. Whites							
IlmnID	Chr	BP position	Gene	Relation to island	Beta	SE	*P*	IlmnID	Chr	BP position	Gene	Relation to island	Beta	SE	*P*
**vcg17268658**	**chr2**	**106015745**	***FHL2***	**Island**	**0.019**	**0.001**	**2.37E−36**	**cg27099280**	**chr15**	**72612204**	***CELF6***	**Island**	**0.044**	**0.004**	**7.19E−26**
**cg27099280**	**chr15**	**72612204**	***CELF6***	**Island**	**0.041**	**0.003**	**1.88E−31**	**cg06210197**	**chr8**	**24771256**	***NEFM***	**Island**	**0.029**	**0.003**	**2.70E−23**
**cg13206721**	**chr10**	**25463350**	***GPR158***	**N_Shore**	**0.023**	**0.002**	**4.91E−31**	**cg26947034**	**chr7**	**33935438**		**OpenSea**	**− 0.020**	**0.002**	**3.43E−21**
**cg26947034**	**chr7**	**33935438**		**OpenSea**	**− 0.022**	**0.002**	**5.28E−27**	**cg24214068**	**chr8**	**24771265**	***NEFM***	**Island**	**0.018**	**0.002**	**4.77E−21**
cg19644580	chr21	19166676	*C21orf91*	OpenSea	**−** 0.016	0.001	6.83E**−**27	**cg12841266**	**chr3**	**9594093**	***LHFPL4***	**Island**	**0.014**	**0.002**	**9.94E−20**
**cg21620282**	**chr14**	**93389628**	***CHGA***	**Island**	**0.023**	**0.002**	**5.93E−26**	**cg07323488**	**chr3**	**168185313**	***EGFEM1P***	**OpenSea**	**− 0.025**	**0.003**	**1.84E−19**
**cg12841266**	**chr3**	**9594093**	***LHFPL4***	**Island**	**0.015**	**0.001**	**1.62E−25**	**cg17268658**	**chr2**	**106015745**	***FHL2***	**Island**	**0.016**	**0.002**	**3.25E−18**
**cg13552692**	**chr18**	**66389447**	***CCDC102B***	**OpenSea**	**− 0.020**	**0.002**	**6.11E−24**	**cg21620282**	**chr14**	**93389628**	***CHGA***	**Island**	**0.020**	**0.002**	**5.44E−18**
**cg07504615**	**chr3**	**14832729**		**OpenSea**	**− 0.014**	**0.001**	**1.44E−22**	cg17740900	chr14	64266659		OpenSea	**−** 0.033	0.004	1.98E**−**17
**cg24214068**	**chr8**	**24771265**	***NEFM***	**Island**	**0.019**	**0.002**	**1.18E−20**	**cg03947688**	**chr5**	**79866382**	***ANKRD34B***	**Island**	**0.017**	**0.002**	**6.05E−17**
**cg12642568**	**chr1**	**1846648**	***CALML6***	**N_Shelf**	**− 0.013**	**0.001**	**1.56E−20**	**cg13206721**	**chr10**	**25463350**	***GPR158***	**N_Shore**	**0.021**	**0.003**	**1.09E−16**
cg14911690	chr19	19729395	*PBX4*	Island	0.021	0.002	7.31E**−**20	**cg13552692**	**chr18**	**66389447**	***CCDC102B***	**OpenSea**	**− 0.021**	**0.003**	**1.53E−16**
cg21165089	chr11	64739736	*C11orf85*	Island	0.014	0.002	1.52E**−**19	**cg02669012**	**chr10**	**58382013**		**OpenSea**	**− 0.023**	**0.003**	**2.24E−16**
cg22116858	chr10	105241934	*NEURL1-AS1*	OpenSea	**−** 0.012	0.001	1.75E**−**19	**cg12642568**	**chr1**	**1846648**	***CALML6***	**N_Shelf**	**− 0.012**	**0.002**	**1.16E−15**
cg12211691	chr1	32211779	*ADGRB2*	S_Shelf	**−** 0.014	0.002	2.43E**−**19	cg27447795	chr1	46309093	*MAST2*	OpenSea	**−** 0.029	0.004	1.28E**−**15
**cg04640687**	**chr13**	**102844744**	***FGF14***	**OpenSea**	**− 0.030**	**0.003**	**3.21E−19**	**cg26081188**	**chr11**	**19726873**	***NAV2***	**OpenSea**	**− 0.017**	**0.002**	**1.70E−15**
**cg21323642**	**chr22**	**31709724**		**OpenSea**	**− 0.015**	**0.002**	**6.31E−19**	cg23565569	chr7	120629502	*CPED1*	OpenSea	**−** 0.025	0.003	2.13E**−**15
cg18758922	chr22	37099785	*CACNG2*	S_Shore	0.017	0.002	1.29E**−**18	**cg24866418**	**chr3**	**9594082**	***LHFPL4***	**Island**	**0.012**	**0.002**	**4.11E−15**
**cg01156568**	**chr6**	**87849235**		**OpenSea**	**− 0.027**	**0.003**	**1.49E−18**	**cg04640687**	**chr13**	**102844744**	***FGF14***	**OpenSea**	**− 0.034**	**0.004**	**5.30E−15**
cg16832267	chr2	18060102	*KCNS3*	Island	0.039	0.005	1.99E**−**18	cg07024568	chr15	71615957	*THSD4*	OpenSea	**−** 0.026	0.003	1.10E**−**14
cg04931708	chr7	121944678	*FEZF1*	S_Shore	0.023	0.003	2.96E**−**18	cg01763090	chr15	31775406	*OTUD7A*	N_Shore	0.017	0.002	1.11E**−**14
**cg26928911**	**chr1**	**22968992**	***C1QC***	**OpenSea**	**− 0.018**	**0.002**	**3.42E−18**	cg21757266	chr13	28896815	*FLT1*	OpenSea	**−** 0.020	0.003	2.29E**−**14
cg24964298	chr1	155959036		OpenSea	**−** 0.012	0.001	3.60E**−**18	cg22353329	chr17	77814357	*CBX4*	Island	0.024	0.003	2.57E**−**14
**cg07132386**	**chr2**	**207139218**	***ZDBF2***	**N_Shore**	**0.024**	**0.003**	**3.82E−18**	cg25273039	chr7	8473512	*NXPH1*	Island	0.015	0.002	3.82E**−**14
**cg24866418**	**chr3**	**9594082**	***LHFPL4***	**Island**	**0.013**	**0.002**	**4.00E−18**	cg08745595	chr1	169556012	*F5*	OpenSea	**−** 0.014	0.002	7.76E**−**14
cg16001722	chr6	127836159	*C6orf174*	Island	0.027	0.003	5.16E**−**18	cg26638716	chr14	80053724	*NRXN3*	OpenSea	**−** 0.025	0.003	8.01E**−**14
cg27037708	chr20	4721316	*PRNT*	OpenSea	**−** 0.017	0.002	5.44E**−**18	**cg12318914**	**chr4**	**17387235**		**OpenSea**	**− 0.026**	**0.003**	**1.28E−13**
cg08637691	chr9	134989631		OpenSea	0.021	0.002	5.48E**−**18	cg11102031	chr6	148060358		OpenSea	**−** 0.015	0.002	1.76E**−**13
**cg02669012**	**chr10**	**58382013**		**OpenSea**	**− 0.019**	**0.002**	**6.34E−18**	cg25181130	chr17	30651431	*RHBDL3*	OpenSea	**−** 0.020	0.003	2.77E**−**13
cg24501701	chr12	63544040	*AVPR1A*	Island	0.013	0.001	9.79E**−**18	cg09461021	chr22	20969574		OpenSea	**−** 0.018	0.002	3.11E**−**13
cg15628888	chr15	72612209	*CELF6*	Island	0.028	0.003	1.08E**−**17	**cg07504615**	**chr3**	**14832729**		**OpenSea**	**− 0.013**	**0.002**	**4.99E−13**
**cg07323488**	**chr3**	**168185313**	***EGFEM1P***	**OpenSea**	**− 0.021**	**0.002**	**2.30E−17**	cg09445550	chr20	5106966	*PCNA*	N_Shore	**−** 0.015	0.002	6.90E**−**13
cg17891166	chr7	73441989	*ELN*	OpenSea	**−** 0.014	0.002	2.87E**−**17	cg06506328	chr10	69373205	*CTNNA3*	OpenSea	**−** 0.021	0.003	7.25E**−**13
**cg04978384**	**chr15**	**58358310**	***ALDH1A2***	**Island**	**0.027**	**0.003**	**4.62E−17**	cg08176240	chr15	31489071		OpenSea	**−** 0.016	0.002	7.42E**−**13
**cg03947688**	**chr5**	**79866382**	***ANKRD34B***	**Island**	**0.015**	**0.002**	**4.68E−17**	cg18805621	chr4	10583796	*CLNK*	OpenSea	**−** 0.018	0.003	1.16E**−**12
**cg12318914**	**chr4**	**17387235**		**OpenSea**	**− 0.026**	**0.003**	**5.64E−17**	cg00067981	chr21	27254444	*APP*	OpenSea	**−** 0.011	0.002	1.44E**−**12
cg22558303	chr13	96705786	*UGGT2*	Island	0.038	0.005	6.53E**−**17	cg17554862	chr2	201991544	*CFLAR*	OpenSea	**−** 0.013	0.002	2.17E**−**12
cg00394316	chr7	22539822	*MGC87042*	Island	0.034	0.004	6.73E**−**17	**cg21323642**	**chr22**	**31709724**		**OpenSea**	**− 0.011**	**0.002**	**2.30E−12**
cg00101260	chr17	53426657		OpenSea	**−** 0.013	0.002	6.81E**−**17	cg16476710	chr14	37304393	*SLC25A21*	OpenSea	**−** 0.026	0.004	2.40E**−**12
cg18284157	chr22	37099802	*CACNG2*	S_Shore	0.017	0.002	8.96E**−**17	**cg04978384**	**chr15**	**58358310**	***ALDH1A2***	**Island**	**0.025**	**0.004**	**2.65E−12**
cg09849302	chr21	38378433	*RIPPLY3*	Island	0.020	0.002	1.73E**−**16	cg16381169	chr21	16031542		OpenSea	**−** 0.014	0.002	2.78E**−**12
cg26153045	chr7	73441834	*ELN*	OpenSea	**−** 0.015	0.002	2.17E**−**16	cg20193323	chr6	7276020		OpenSea	**−** 0.027	0.004	3.21E**−**12
**cg26081188**	**chr11**	**19726873**	***NAV2***	**OpenSea**	**− 0.017**	**0.002**	**2.42E−16**	cg16169166	chr3	14303148		OpenSea	**−** 0.021	0.003	3.36E**−**12
cg21328810	chr19	50829227	*KCNC3*	Island	0.013	0.002	2.59E**−**16	cg10934519	chr3	69720954		OpenSea	**−** 0.015	0.002	3.99E**−**12
cg25258740	chr12	75603230	*KCNC2*	Island	0.032	0.004	2.60E**−**16	cg17365308	chr12	126536415		OpenSea	**−** 0.020	0.003	5.40E**−**12
cg07522171	chr7	28218686	*JAZF1-AS1*	N_Shore	**−** 0.015	0.002	2.92E**−**16	cg26772387	chr15	91846753		OpenSea	**−** 0.013	0.002	5.96E**−**12
cg20168635	chr15	83916827		OpenSea	**−** 0.017	0.002	4.28E**−**16	**cg26928911**	**chr1**	**22968992**	***C1QC***	**OpenSea**	**− 0.016**	**0.002**	**7.05E−12**
cg25045526	chr7	121944506	*FEZF1*	Island	0.019	0.002	4.84E**−**16	cg09579833	chr3	49849871	*UBA7*	OpenSea	**−** 0.013	0.002	7.37E**−**12
cg26079664	chr3	51740956	*GRM2*	Island	0.028	0.003	5.18E**−**16	**cg07132386**	**chr2**	**207139218**	***ZDBF2***	**N_Shore**	**0.018**	**0.003**	**8.17E−12**
**cg06210197**	**chr8**	**24771256**	***NEFM***	**Island**	**0.023**	**0.003**	**5.29E−16**	**cg01156568**	**chr6**	**87849235**		**OpenSea**	**− 0.022**	**0.003**	**1.15E−11**

*IlmnID*: CpG probe identification number, *SE:* standard error

*Overlapping age-associated differentially methylated CpG positions between African Americans and whites are highlighted in bold

### Genomic feature enrichment and functional annotations

We then characterized each of the significant aDMPs for enrichment across various regulatory regions of the genome as determined by the ENCODE and FANTOM projects using Fisher’s exact test. Enrichment analysis was performed separately for aDMPs that showed gain (hypermethylated) and loss of methylation (hypomethylated) with chronological age. As expected, aDMPs that gained methylation with age were enriched at CpG islands (Additional file 4: Table S2). Hypermethylated aDMPs were also enriched at 5′ UTR, DNase I hypersensitivity sites (DHS), first exon regions, and reprogramming-specific genomic regions (Additional file 4: Table S2). Hypomethylated aDMPs were enriched at Open Sea regions, transcription factor binding sites, CpG island shore regions, and open chromatin regions (Additional file 5: Table S3).

To further understand the functional significance of aDMPs and to identify canonical pathways overrepresented among aDMPs, we performed gene ontology (GO) enrichment and Kyoto Encyclopedia of Genes and Genomes (KEGG) pathway analysis while accounting for the differences in the number of CpG positions present in each gene in the MethylationEPIC BeadChip. aDMPs in both AAs and whites were enriched for gene ontology terms related to system and organismal development and morphogenesis. These top terms include central nervous system development, multicellular organism development, and cell-cell signaling (Additional file 6: Table S4 and Additional file 7: Table S5). These results suggest that aging may affect the methylation status of genes and pathways that are important for growth and development in the nervous system and other organ systems.

### Age-related disease gene enrichment analysis

To characterize the significance of hyper- and hypomethylated aDMPs in age-related diseases and phenotypes, we performed enrichment analysis using genes identified by genome-wide association studies (GWAS). We focused on GWAS-identified genes implicated in age-related diseases and quantitative traits and longevity and survival (overall and disease-specific) [38]. After accounting for multiple testing, we found that genes containing hypermethylated aDMPs were enriched for genes linked with visceral fat distribution, lung function, cognitive ability, blood pressure, and IgG glycosylation (Additional file 8: Table S6). Hypomethylated aDMPs were enriched for iron homeostasis and breast cancer (Additional file 9: Table S7).

### Assessment of DNA methylation age using the epigenetic clock

**Fig. 3:**
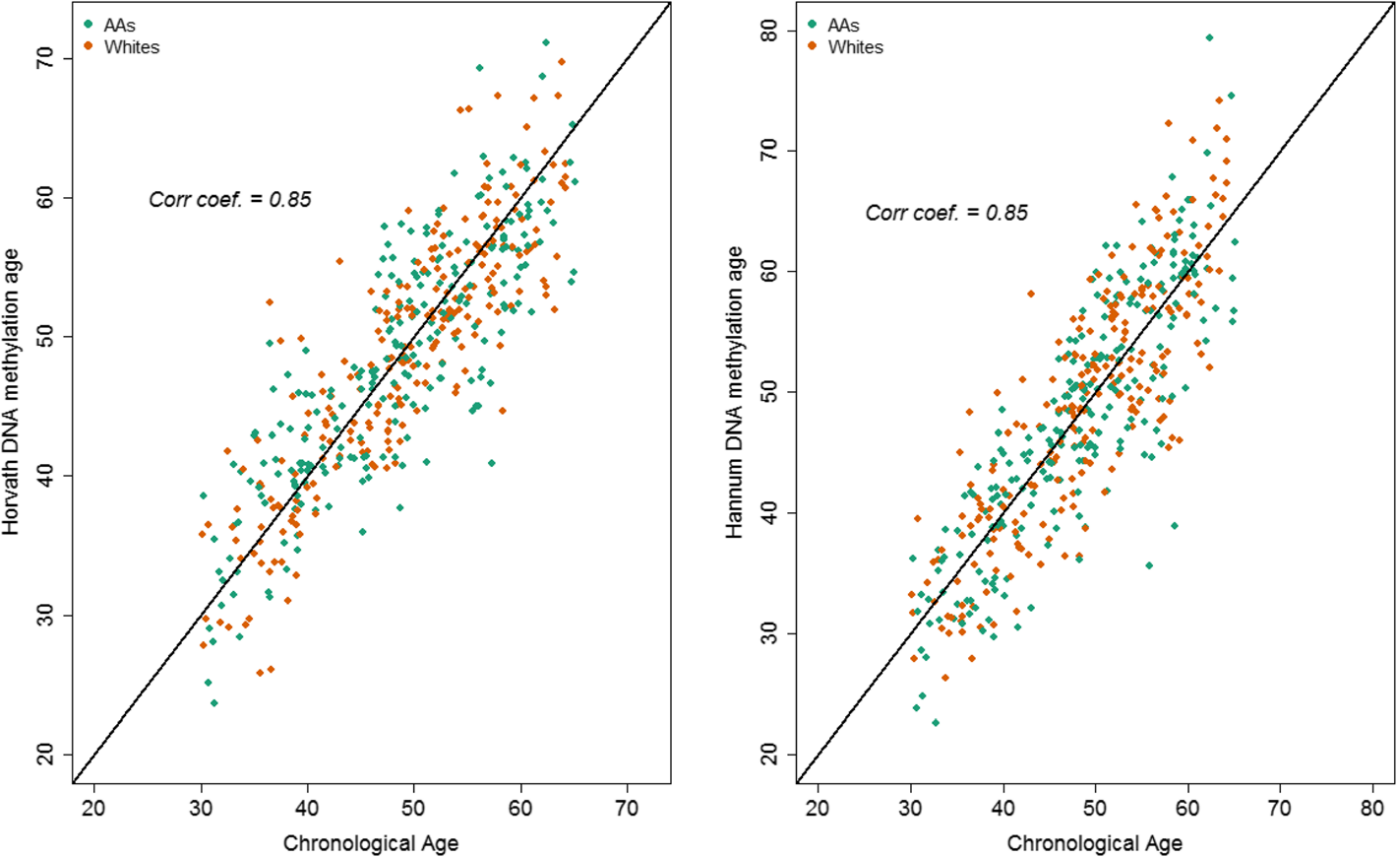
Correlation between DNA methylation-predicted age based on the Horvath and the Hannum clocks, and chronological age in the HANDLS study. Abbreviation: AAs: African Americans

We calculated DNA methylation age (DNAm age) for each of the participants using the Horvath [24] and Hannum algorithms [25] implemented in the online DNAm age calculator [24]. DNAm age predicted by both the Horvath and the Hannum clock was strongly correlated with chronological age (Pearson’s *r* = 0.85) (Fig. 3). In subgroup correlation analysis stratified by sex, race, and poverty status, similar strong correlations between chronological age and DNAm age were observed (Pearson’s *r* range 0.83–0.89) indicating that the epigenetic clocks are robust estimator of chronological age and that the prediction algorithms performed well in our cohort.

### Determinants of epigenetic age acceleration

**Fig. 4:**
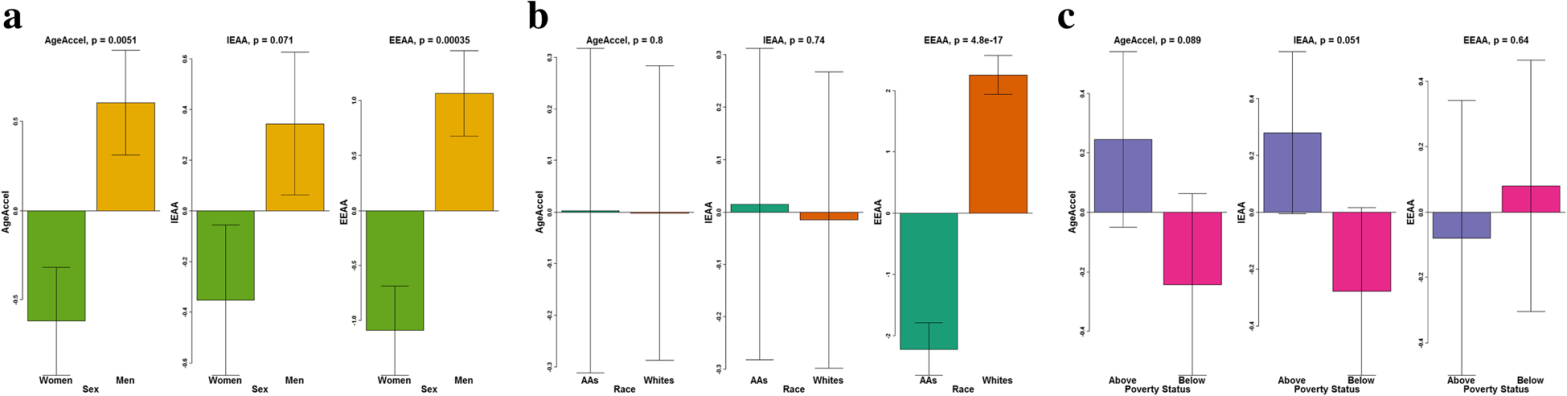
Associations between epigenetic age acceleration measures and sex, race, and poverty status. **a** Sex, **b** race, and **c** poverty status. Abbreviations: AAs: African Americans; AgeAccel: universal age acceleration measures; IEAA: intrinsic epigenetic age acceleration; and EEAA: extrinsic epigenetic age acceleration

Epigenetic age acceleration, generally defined as the difference between DNAm age and chronological age, has been suggested as a promising marker of biological age [31]. We hypothesized that there would be population differences in biological age such that men compared to women, AAs compared to whites, and individuals below poverty status compared to those above would be fast agers. To test our hypothesis, we first computed a universal measure of age acceleration (AgeAccel) as the residuals of regressing DNAm age predicted by the Horvath method, which is independent of cell and tissue types, over chronological age. The absolute mean (± standard error) AgeAccel (in years) was − 1.03 (± 0.47) in AA women, + 1.01 (± 0.40) in AA men, − 0.20 (± 0.19) in white women, and + 0.19 (± 0.43) in white men. Positive residual values of AgeAccel indicate faster aging based on chronological age, and negative residual values indicate slower aging. Two additional measures of epigenetic age acceleration were derived: intrinsic epigenetic age acceleration (IEAA) and extrinsic epigenetic age acceleration (EEAA). IEAA is meant to capture cell-intrinsic properties of the aging process, which is independent of estimated white blood cell type proportions, while the EEAA measure is enhanced by white blood cell estimates and may further capture age of the immune system cells (immunosenescence) [31]. We found that men compared to women were fast agers in AgeAccel and EEAA (Fig. 4a). No differences between AAs and whites were observed in AgeAccel and IEAA measures, but significant differences in EEAA were observed by race (*p* = 4.8E−17) (Fig. 4b). Poverty status was not associated with any of the epigenetic age acceleration measures (Fig. 4c).

**Fig. 5:**
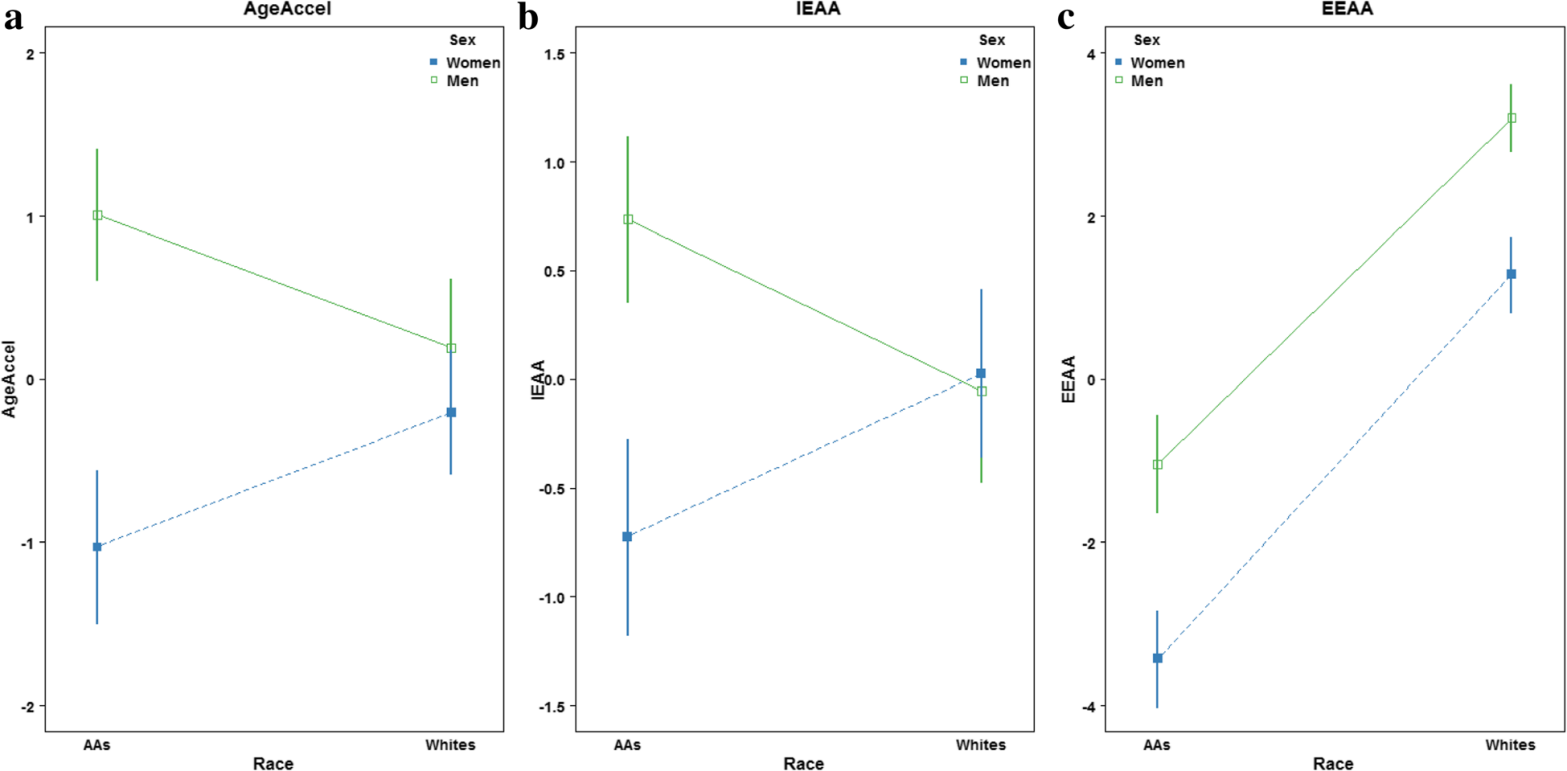
Interaction plots of the association between sex, race, and three measures of epigenetic age acceleration. **a** AgeAccel, **b** IEAA, and **c** EEAA. Abbreviations: AAs: African Americans; AgeAccel: universal age acceleration measures; IEAA: intrinsic epigenetic age acceleration; and EEAA: extrinsic epigenetic age acceleration

We used linear regression models to assess two-way interactions between sex, race, and poverty status on the three epigenetic age acceleration measures. A nominally significant *p* value between sex and race for AgeAccel (*p*_interaction-term_ = 0.049) was observed but not for IEAA (*p*_interaction-term_ = 0.058) or EEAA (*p*_interaction-term_ = 0.6). Specifically, AA men by + 2.04 years (*p* = 6.07E−04) and white men by + 1.24 years (*p* = 0.038) were fast agers using AgeAccel compared to AA women (Table 3). However, if we were to account for multiple testing (nine tests: three age acceleration measures and three two-way interaction terms), the results become non-significant. Figure 5a–c shows interaction plots of the association between sex, race, and AgeAccel, IEEA, and EEAA and highlights the AgeAccel differences between AA women, AA men, and white men.

**Table 3:** Association between sex, race, poverty status, and measures of epigenetic age acceleration

Parameters	*N*	AgeAccel			IEAA			EEAA		
		Beta	SE	*P*	Beta	SE	*P*	Beta	SE	*P*
Race, sex										
African American, women	117	Ref.			Ref.			Ref.		
African American, men	120	2.04	0.59	*6.07E−04*	1.47	0.58	*0.011*	2.39	0.74	*1.25E−03*
White, women	115	0.84	0.60	0.16	0.76	0.58	0.2	4.72	0.75	*5.48E−10*
White, men	118	1.24	0.59	*0.038*	0.69	0.58	0.2	6.64	0.74	*7.48E−18*
*P* interaction				*0.049*			0.058			0.6
Sex, poverty status										
Women, above poverty	117	Ref.			Ref.			Ref.		
Women, below poverty	115	− 0.52	0.60	0.4	− 0.51	0.58	0.4	− 0.42	0.74	0.6
Men, above poverty	117	1.21	0.60	*0.04*	0.76	0.58	0.2	1.69	0.74	*0.02*
Men, below poverty	121	0.73	0.59	0.2	0.14	0.58	0.8	2.20	0.73	*0.003*
*P* interaction				0.97			0.9			0.4
Race, poverty status										
African American, above	120	Ref.			Ref.			Ref.		
African American, below	117	− 0.81	0.59	0.2	− 0.90	0.58	0.1	− 0.42	0.74	0.6
White, above	114	− 0.31	0.60	0.6	− 0.36	0.58	0.5	4.00	0.74	*1.11E−07*
White, below	119	− 0.50	0.59	0.4	− 0.58	0.57	0.3	4.54	0.73	*1.34E−09*
*P* interaction				0.5			0.4			0.4

*AgeAccel* universal epigenetic age acceleration, *EEAA* extrinsic epigenetic age acceleration, *IEAA* intrinsic epigenetic age acceleration, *SE* standard error

## Discussion

To the best of our knowledge, this is the first study to apply the MethylationEPIC BeadChip with its enhanced and expanded genome-wide CpG coverage to assess the genome-wide distribution of age-associated DNA methylation changes and to perform comparative analysis of aDMPs among socioeconomically diverse urban community-dwelling AAs and whites. We replicated several aDMPs previously discovered in blood DNA using the 27K and 450K arrays. However, we identified novel CpGs (75.6% of significant aDMPs in AAs and 53.1% of significant aDMPs in whites) with small effect sizes that were either hyper- or hypomethylated with age using stringent significance threshold criteria. Interestingly, we found that compared to whites, AAs display more widespread DNA methylation changes. Our results also indicate that, compared to AA women, white men and AA men are found to be fast agers as indicated by AgeAccel and IEAA measures. On the other hand, AA women have a slower age of the immune system cells as indicated by EEAA. Contrary to our hypothesis, there is no association between poverty status and any of the epigenetic age acceleration measures.

Age and the social determinants of health (sociodemographic characteristics, lifestyle, and environmental factors) are important risk factors of most chronic diseases. In many ways, these risk factors disproportionately affect racial minorities, socioeconomically disadvantaged, and marginalized population groups. There is ample evidence in the literature that supports the notion of premature aging or “weathering” among AAs in particular [39]. How these social determinants of health bring about the molecular and cellular changes that lead to chronic disease and aging-related health disparities is poorly understood. DNAm is one of the epigenetic modifications that plays an important role in the regulation of various cellular processes including developmental processes and imprinting, gene expression, and maintenance of genome stability [4]. DNAm, which is a dynamic process that is continuously added and removed from the genome during the lifespan of an individual, is affected by both environmental exposures and external stresses. Therefore, it could play a role in age-related health disparities. Aging is generally characterized by DNAm changes specifically a gain of methylation in CpG islands, bivalent chromatin domains [21], and polycomb-group target genes [22], and loss of methylation predominantly in non-CpG island regions and in the active chromatin mark H3K4me1 [11]. In regard to CpGs used in the epigenetic clocks, there is evidence in AAs indicating that a third of the CpGs in the Horvath’s epigenetic clock respond to glucocorticoid receptor activation and influence the gene expression of stress-responsive genes which are enriched for association with aging-related diseases [40]. Further, some of the CpGs found in the epigenetic clock of mice have been shown to be involved in the development, differentiation, and tissue morphogenesis consistent with a program-like behavior [41]. Although the exact mechanisms that drive changes in DNAm during aging are not fully understood, by the virtue of its link with age-related diseases and risk factors, DNAm is a promising molecular factor that could link health disparities and its risk factors. Therefore, understanding age-related DNAm changes and identification of differential association among racial groups could shed light on aging and aging-related health disparities.

Our findings of aDMPs and their enrichment for genomic regulatory elements, developmental, and morphogenesis processes are broadly consistent with previously published epigenetic association studies of chronological age showing that age is associated with extensive DNA methylation changes (both hyper- and hypomethylation) that overlapped with functional genomic regulatory regions [11, 21, 22]. We observed that genes containing aDMPs overlap with genes implicated in several age-related diseases and traits in genome-wide studies [38]. This overlap between genes containing aDMPs and age-related disease genes and traits implies there are common factors and underlying mechanisms that generally control changes in DNAm with age and the development of age-related diseases. Understanding these factors and the underlying molecular mechanisms will have implications in the effort to narrow down the age-related disease disparity gap between population groups. We replicated several aDMPs including those located in *ELOVL2*, *PENK*, *KLF14*, and *SLC12A5*. Interestingly, methylation changes in *ELOVL2* and *PENK* are associated with age in various tissues in addition to DNA derived from blood [25, 27] suggesting that some age-associated methylation changes are tissue independent and that blood DNA could be used to further investigate the underlying biological mechanisms and downstream functional alterations in large population-based studies where blood is the most feasible source of DNA for methylation profiling. *ELOVL2* has been linked to changes in human plasma metabolite levels such as glycerophospholipids [42], and photoaging response in epidermis [43]. It remains to be seen how age-associated DNAm changes in *ELOVL2* and the other consistently replicated genes mechanistically contribute to aging-related disease development and accelerated biological aging phenotypes. The epigenome-wide association analysis results of methylation M values are reported here (Additional file 11: Figure S4).

The aDMPs we identified, similar to previous studies, have small beta coefficients, consistent with previous observations with other traits and exposures. What is the biological meaning of these large numbers of aDMPs with small effect sizes? First, one of the functions of DNA methylation is regulation and fine tuning of transcription by transducing external and internal cues, and hence, large effects may not be observed. Second, age-related common diseases are multifactorial in origin and include genetic and epigenetics factors which act in concert with each other or other risk factors. Therefore, it is possible that there could be interactions between significantly associated aDMPs and acting in synergy (between themselves or with other epigenetic modifications such as histone acetylation and methylation); they could bring about changes in transcription thereby contributing multiplicatively to age-related disease risk. Third, accumulating evidence indicates that these age-related chronic diseases have been shown to be associated with a large number of genetic sites mostly with small effects. Epigenetics and epigenetic inheritance have been put forward as a potential explanation for missing heritability of complex diseases, i.e., inherited risk factors of common complex diseases and traits that are yet to be identified [44]. Therefore, CpGs with small effect sizes would be consistent with the observations that common complex age-related diseases are associated with a large number of sites with small effect sizes that could have cumulative effect on disease risk. Finally, analogous to the variety of different molecular and cellular changes such as mutations that accrue over the life course of an individual, it is possible that not all the DNA methylation changes linked with age may have discernable biological consequences, and they might be “passengers” rather than key “drivers” DNA methylation changes that could lead to the expression a certain aging-related trait or disorder [45]. While these explanations require empirical data, it is important to note that large numbers of CpGs with small effect sizes linked with age, several environmental exposures, and other traits have been reported in diverse study designs and settings and were consistently replicated suggesting that these seemingly small effect size associations are robust and could have true biological significances [46].

Our findings that men are generally fast agers as indicated by AgeAccel and EEAA measures are consistent with previous studies of age acceleration studies using DNA extracted from blood, brain, and saliva [34, 35, 47]. This biological age acceleration difference between men and women has been postulated to explain the sex morbidity-mortality paradox. Several factors have been put forward to explain this observed difference including differences in health seeking behavior and lifestyle factors [34]. The slower immune system cell age seen in AAs compared to whites is consistent with studies that reported longer leukocyte telomere length in AAs compared to whites [48, 49].

It should be noted that not all aging-related diseases are associated with the premature aging phenotype. For example, cancer tissues have been shown to display inconsistent patterns of aging rate which is dependent on the site of cancer origin, cancer stage/histology, and type and the number of driver somatic mutations. Compared to adjacent normal tissue, slower epigenetic age was observed in basal-like breast cancer and glioblastoma multiforme with *H3F3A* mutations [24]. On the one hand, faster epigenetic age was observed in tumors carrying a smaller number of somatic mutations, tumors with *TP53* mutations, acute myeloid leukemia, hormone receptor-positive (luminal type) breast cancer, and BRAF-positive colorectal cancer [24, 50]. In addition, faster epigenetic age in various cancer tissue samples (lung, skin, breast, and kidney), compared to matched normal tissue samples, has also been reported [25].

The observation of a faster epigenetic aging in AA men is consistent with the epidemiologic literature reporting higher chronic disease risk earlier in the life course of AA men and premature mortality rates [1, 2, 51]. This finding implies that the biological aging rate of AA men ticks faster before they succumb to age-related diseases. What is driving this observed age acceleration in AA men? Stressors (psychosocial and socioeconomic stress and their correlates) could be one potential explanation. Although we did not observe in our study differential association between poverty status and any of the epigenetic age acceleration measures, cumulative lifetime stress has been shown to cause epigenetic age acceleration in AAs possibly through glucocorticoid-induced epigenetic changes [40].

The implication of our study is that some of aDMPs and the epigenetic clock could be utilized for the identification of at-risk groups or to determine the efficacy of clinical and public health interventions to extend lifespan and reverse the accelerated aging process. Recent studies conducted in animal models provide a potential usefulness of these age-associated DNAm changes in identifying interventions to extend longevity. Using mouse models and non-human primate studies, Maegawa et al. showed that age-associated DNAm changes are amenable to longevity intervention. Specifically, they found that mice and rhesus monkeys exposed to caloric restriction showed attenuation of age-associated methylation changes compared to ad libitum-fed controls such that their blood DNAm age appeared younger than their chronologic age, and these effects were detectable across different tissues [52]. Another study by Petkovich et al. conducted in mouse models showed that the epigenetic clock accurately estimates biological age of various mouse models, and it could be used to evaluate the longevity effects of caloric restriction, pharmacological interventions, and genetic intervention of longevity such as growth hormone receptor knockout [41]. These results suggest that anti-aging interventions can affect the epigenetic clock in mouse and in non-human primates, and future research lies in determining whether these findings translate to humans.

Our study has a number of strengths: (1) large sample size, (2) use of MethylationEPIC array that doubled the number of genome-wide CpG sites to interrogate and improve one of the limitations of previous epigenome-wide association studies of age, and (3) study participants with diverse characteristics and balanced sample size. The limitations of our study include the use of DNA derived from mixed peripheral blood cells, which could confound the observed association between CpG positions and chronological age. To address the issue of confounding due to cellular heterogeneity, we included white blood cell estimates based on DNAm in our regression models. While studying DNAm changes in DNA derived from sorted white blood cells is ideal, it is not scalable in large population-based studies like ours. Other limitations include the use of cross-sectional data and lack of validation sample to confirm our race-specific aDMPs. Because of the cross-sectional nature of our study, it is impossible in this design to determine whether all the reported aDMPs in our study were directly driven by age. In addition, it must be considered that it is possible that the high number of significant sites could also be influenced by population sub-stratification and other unmeasured covariates. Functional studies in experimental model systems of aging and age-related diseases could provide further biological insight and help interpret these results.

## Conclusions

In summary, we found that age differences are associated with DNAm changes at several genes enriched for predicted functional genomic regulatory regions and that AAs compared to whites have more aDMPs, majority of which are novel CpG sites. The identification of novel aDMPs has the potential to expand our knowledge of the effect of age on DNAm and its differential effect among racial groups. Our study also shows that there is an interaction between sex and race in influencing epigenetic age acceleration among population groups. These age-associated genes could provide insight in the epigenetic bases of aging and age-related health disparities and could explain the observed differences in disease incidence and lifespan between AAs and whites. Future larger studies with longitudinal data are required to replicate our findings. The results generated in the present study also provide a valuable resource to the study and prioritization of genes and gene networks that might be implicated in aging and age-related diseases, and to advance the nascent field of the epigenetics of health disparities.

## Methods

### Study aim, design, and population

Participants were drawn from the HANDLS study (https://handls.nih.gov/) [36], a large population-based prospective longitudinal study of middle-aged AA and white men and women above and below poverty status being conducted in Baltimore, Maryland. HANDLS was designed to explore the interplay among sex, race, poverty status, and biological and environmental factors in the development of aging-related diseases and health disparities in community-dwelling adults vulnerable for health disparities. Participants eligible for this study had DNA samples isolated from blood at their enrollment. We randomly sampled 508 participants using a factorial design across sex, race, and poverty status, oversampling AA men below poverty status, to test for interactions among these sociodemographic factors. From these samples, 487 had DNA methylation measures. The age range at baseline was 30.2 to 65.2 years [mean age (standard deviation) = 47.8 (8.7)]. Poverty status in the HANDLS study was defined as a household income above or below 125% of the 2004 US Federal Poverty Guidelines, and race was self-identified. We used blood DNA and sociodemographic data collected during the enrollment period, from 2004 to 2009. DNA was extracted from peripheral blood mononuclear cells using standard methods.

### Bisulfite treatment and DNA methylation quantification using the EPIC BeadChip

Two hundred fifty nanograms of DNA was treated with sodium bisulfite using Zymo EZ-96 DNA Methylation kit as per the manufacturer’s protocol (Zymo Research, Orange, CA, USA). Following bisulfite treatment, we then measured genome-wide DNAm using the latest Illumina Infinium MethylationEPIC BeadChip, which contains 866,836 CpG sites, of these, 142,262 (16.4%) were assayed in type I probes and the rest in type II probes. This new EPIC array has similar Infinium assay design chemistry as the previous 450K array, except that it has twice as much coverage of CpG sites that are particularly enriched for promoter and enhancer regulatory regions [53], providing increased power and genome coverage to identify novel loci relevant for aging and health disparities. The EPIC array contains 92% and 94% of CpG sites found in the 27K and 450K arrays, respectively. We included 12 technical replicates (two per plate) for quality control.

### Quality control and preprocessing of the DNA methylation data

We performed extensive quality control of the DNAm data at sample and probe levels to ensure high-quality methylation data. Given the EPIC array is a new technology, we assessed the performance of different data normalization and preprocessing algorithms in reducing technical variations using DNA methylation measured in technical replicates to identify a suitable method. The methods we compared were Illumina Genome Studio, normal-exponential out-of-band (NOOB) [54], stratified quantile normalization (quantile) [55], and subset-quantile within array normalization (SWAN) [56]. At the sample level, we excluded 17 samples which were multidimensional scaling outliers, low-quality methylation values as indicated by a mean detection *p* value ≥ 0.01 and have evidence of sex mismatch between self-reported sex and methylation predicted sex. At the probe level, we excluded low-quality probes (mean detection *p* value ≥ 0.01), probes with overlapping single-nucleotide polymorphisms (SNPs) (minor allele frequency cutoff = 0.05), cross-hybridizing probes [37], and probes mapping to the sex chromosomes leaving 765,808 CpG positions for the current analysis.

To identify an optimal method for normalization, we compared the performance of the above methods using correlations and probe variances of methylation beta values of technical replicates. We found the NOOB method yielded the lowest variance and highest correlation between technical replicates. Therefore, this method was used to normalize the EPIC methylation data in our cohort. Regression on correlated probes (RCP) method was used to correct for type I and type II probe design biases [57]. We performed principal component analysis to identify the presence of experimental batch effect and beadchip position. We then applied the *ComBat* method to adjust for batch effect [58]. We estimated white blood cell proportions based on DNAm data using the Houseman and the Horvath methods [24, 59]. The Houseman method estimated the proportion of CD8^+^ and CD4^+^ T lymphocytes, natural killer cells, B lymphocytes, monocytes, and granulocytes. The Horvath method was used to estimate the percentage of exhausted CD8^+^ T cells (CD8^+^CD28^−^CD45RA^−^), plasmablasts, and the number of naïve CD8^+^ T cells (CD8^+^CD45RA^+^CCR7^+^).

### Principal component analysis and control for population stratification

To correct for population stratification, we calculated methylation-based principal components. Barfield et al. showed that principal components calculated based on CpG positions that overlap with SNPs serve as proxy and provide powerful and computationally efficient approach to account for population stratification in the absence of genetic data [60]. We used the methylation level of 7905 CpG positions that passed quality control and overlapped with SNPs to calculate principal components. The first two principal components were then in the regression models to control for population stratification. Once these CpG positions were used for the calculation of principal components, they were dropped before downstream analysis.

### DNA methylation age prediction and epigenetic age acceleration measures

DNAm age was calculated using the Horvath and the Hannum methods based on the methylation levels of 353 and 71 CpG sites, respectively, using the epigenetic clock algorithm. The algorithms were trained and validated on participants from varied genetic ancestries and using DNA derived from various tissues including blood DNA. A detailed description of estimation of DNAm age and epigenetic age acceleration can be found here: (https://labs.genetics.ucla.edu/horvath/dnamage/). Briefly, the Horvath method predicts age irrespective of the tissue or cell source of DNA, and hence, it is tissue and cell type agnostic. On the other hand, the Hannum method was developed based on blood DNAm. Universal epigenetic age acceleration (AgeAccel) is defined as the residuals after regressing DNAm age predicted by the Horvath algorithm over chronological age. A positive residual value suggests a faster aging, and a negative value suggests a slower aging. In addition to the AgeAccel measure, we used two additional epigenetic age acceleration measures that reflect intrinsic and extrinsic epigenetic age acceleration—IEAA and EEAA, respectively. IEAA is thought to measure a cell’s epigenetic age acceleration, independent of estimated white blood cell type proportions. It is defined as the residuals after regressing DNAm age (predicted by the Horvath over chronological age and white blood cell proportions (naive CD8^+^ T cells, exhausted CD8^+^ T cells, plasmablasts, CD4^+^ T cells, natural killer cells, monocytes, and granulocytes). EEAA, which is based on the DNAm age (predicted by the Hannum algorithm), is thought to measure epigenetic age acceleration in addition to changes in white blood cell proportions, and it might be indicative of immune system cell aging (immunosenescence) [31]. Chen et al. showed that EEAA strongly predicts mortality better than the other measures of epigenetic age acceleration [31].

### Epigenome-wide association study of age

To identify CpG positions associated with chronological age in AAs and whites, we fitted linear regression models adjusted for sex, race, poverty status, white blood cell compositions (granulocytes, monocytes, natural killer cells, B lymphocytes, CD4^+^, and CD8^+^ T lymphocytes), and principal components to account for population stratification. The resulting epigenome-wide results were adjusted using estimated empirical null distribution method, a recently described method for controlling genomic inflation and bias in epigenome-wide association studies [61].

The quantile-quantile plots, histogram and density plots of *p* values, and corresponding inflation measures before (AA_inflation_ = 1.040 and whites_inflation_ = 1.263) and after (AA_inflation_ = 0.998 and whites_inflation_ = 0.807) correction for inflation in both AAs and whites are shown in Additional file 10: Figure S3. In order to confirm the approximately 5000 differences found after correcting for genomic inflation were valid, we performed a sensitivity analyses by testing associations based on winsorized DNA methylation data. To increase the power estimation, we performed the winsorization for the extreme values over 3*IQR.

The inflation correction was performed using an R/Bioconductor package BACON [61], which constructs an empirical null distribution using a Gibbs Sampling algorithm by fitting a three-component normal mixture on *z*-scores. Respective genomic inflation factor (lambda) values before and after the correction were provided in the Additional file 12: Table S8. We used the DNAm beta (*β*) and *M* values of 765,808 CpG positions that passed quality control in the regression analysis. The DNAm *β* values were calculated as the ratio of the methylated (*M*) and unmethylated (*U*) fluorescent intensity signals, i.e., *β* = *M*/[*M* + *U* + 100], where 0 indicates unmethylated and 1 indicates fully methylated status. The methylation *M* value was defined as *M* = logit(*β*) and was shown to approximate a normal distribution and provide good model fit [62]. A Bonferroni corrected *p* value of 6.53E–08 was used as a significance threshold. We excluded CpG with effect size between − 0.01 and 0.01. The summary statistics are available at the HANDLS study website (https://handls.nih.gov/). The lambda estimates for *M* values after the inflation correction for AAs ranged from 0.99 to 1.39, whereas for whites, it ranged from 0.80 to 1.21 (Additional file 12: Table S8).

### Genomic feature enrichment and functional annotations

Genomic regulatory feature enrichment was performed on significant aDMPs using one-sided Fisher’s exact test. The genomic regulatory regions compared for enrichment analysis included CpG islands, shelves, shores, DNase I hypersensitivity sites, open chromatin states, exon boundaries, transcription factor binding sites, and Phantom enhancer regions. Functional characterization through gene ontology and canonical pathway analysis was performed by accounting for the differing number of methylation probes found in each gene in the EPIC array given the evidence that a severe bias exists when performing gene set analysis for genome-wide methylation data that occurs due to the differing numbers of CpG sites profiled for each gene [63].

### Age-related disease gene enrichment analysis

To assess enrichment of genes containing aDMPs for various age-related diseases and quantitative phenotypes, we leveraged the genome-wide association study (GWAS) summary association statistics catalog of various age-related diseases and traits available at the National Human Genome Research Institute (NHGRI) and the European Molecular Biology Laboratory (EMBL) [38]. Using the summary statistics catalog (accessed on July 17, 2018, https://www.ebi.ac.uk/gwas/) that included gene names, ontology annotations, and diseases/traits, we investigated whether genes containing aDMPs were overrepresented in GWAS-identified age-related diseases genes. While age is associated with many diseases and their risk factors and quantitative traits, the enrichment analysis was focused on GWAS-identified genes implicated in age-related diseases and traits, longevity, and survival (overall and disease-specific). Enrichment analysis was performed separately for hyper- and hypomethylated aDMPs using one-sided Fisher’s exact test.

### Association between epigenetic age acceleration and demographic characteristics

Linear regression models adjusted for sex, race, and poverty status were used to identify associations between the epigenetic age acceleration measures (AgeAccel, IEAA, and EEAA) and these factors. We also assessed all two-way interactions among sex, race, and poverty status on the epigenetic age acceleration measures. Data quality control, preprocessing, and data analyses were conducted using the R/Bioconductor packages *minfi*, *enmix*, *dmrcate*, *sva*, and *missmethyl* (https://www.bioconductor.org/).

## Additional files

Additional file 1: **Figure S1.** Comparison of the performances of different DNA methylation data normalization and preprocessing methods: (a) correlation between technical replicates; (b) probe variance between technical replicates. (PPTX 100 kb)

Additional file 2: **Table S1.** List of previously discovered age-associated differentially methylated CpG positions (aDMPs) replicated in the HANDLS study. (XLSX 135 kb)

Additional file 3: **Figure S2.** Scatter plots and Pearson’s correlation coefficients of methylation *M* values of top ten age-associated differentially methylated CpG positions (aDMPs) and chronological age in (a) African Americans and (b) whites. (PPTX 121 kb)

Additional file 4: **Table S2.** Genomic feature enrichment of significantly age-associated differentially methylated CpG positions (aDMPs)—hypermethylated. (XLSX 10 kb)

Additional file 5: **Table S3.** Genomic feature enrichment of significantly age-associated differentially methylated CpG positions (aDMPs)—hypomethylated. (XLSX 10 kb)

Additional file 6: **Table S4.** Gene-set enrichment analysis of age-associated differentially methylated CpG positions (aDMPs). (XLSX 11 kb)

Additional file 7: **Table S5.** KEGG pathway analysis of age-associated differentially methylated CpG positions (aDMPs). (XLSX 11 kb)

Additional file 8: **Table S6.** Top enrichment results of age-associated differentially hypermethylated CpG positions (aDMPs) for age-related diseases and quantitative traits. (XLSX 12 kb)

Additional file 9: **Table S7.** Top enrichment results of age-associated differentially hypomethylated CpG positions (aDMPs) for age-related diseases and quantitative traits. (XLSX 8 kb)

Additional file 10: **Figure S3.** Quantile-quantile plots of expected and observed *p* values of the association between DNA methylation and chronological age in African Americans (green) and whites (magenta) A) uncorrected and B) corrected using empirical null distribution. Genomic inflation measures are shown in parentheses. Histogram of test statistic expected (green) and observed (red) *p* values of the association between DNA methylation and chronological age in African Americans and whites C) uncorrected and D) corrected using empirical null distribution. Density plot of expected (black) and observed (red) *p* values of the association between DNA methylation and chronological age in African Americans and whites E) uncorrected and F) corrected using empirical null distribution. (PPTX 234 kb)

Additional file 11: **Figure S4.** Volcano plots showing the distribution of age-associated differentially methylated CpG positions (aDMPs) with their effect size in *M* values and significance *p* value in the a) African American (AA) and b) white participants of the HANDLS study. (PPTX 197 kb)

Additional file 12: **Table S8.** List of genomic inflation factor lambda values calculated for African American (AA) and white population. (XLSX 10 kb)

## Data Availability

The data that support the findings of this study are available from the corresponding author upon reasonable request. The full summary statistics of the epigenome-wide association study of age in AAs and whites are available at the study’s website (https://handls.nih.gov/).

## Abbreviations

AAs: African Americans
aDMPs: Age-associated differentially methylated CpG positions
AgeAccel: Universal measure of epigenetic age acceleration
DNAm age: DNA methylation age (epigenetic age) predicted by the epigenetic clock
DNAm: DNA methylation
EEAA: Extrinsic epigenetic age acceleration
HANDLS: Healthy Aging in Neighborhoods of Diversity across the Life Span
IEAA: Intrinsic epigenetic age acceleration
