# Supplementary material for: Novel age-associated DNA methylation changes and epigenetic age acceleration in middle-aged African Americans and whites
Source: Clin Epigenetics. 2019 Aug 19;11:119. doi: 10.1186/s13148-019-0722-1 (PMC6700815; doi:10.1186/s13148-019-0722-1)
Supplement: Supplementary file 1 — Figure S1. Comparison of the performances of different DNA methylation data normalization and preprocessing methods: (a) correlation between technical replicates; (b) probe variance between technical replicates. (PPTX 100 kb) [file 13148_2019_722_MOESM1_ESM.pptx]

## Slide 1
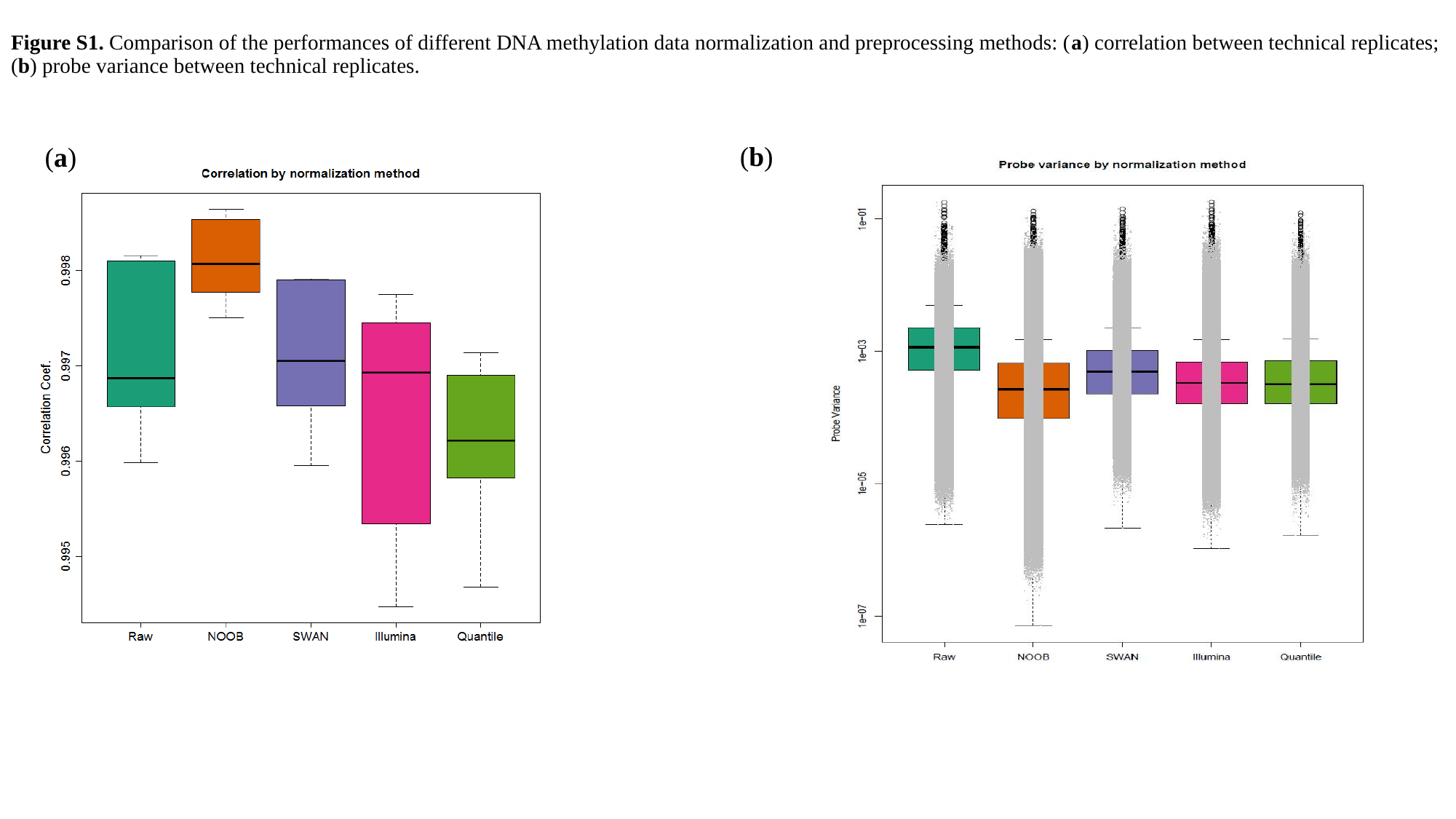

# Figure S1. Comparison of the performances of different DNA methylation data normalization and preprocessing methods: (a) correlation between technical replicates; (b) probe variance between technical replicates.
(b)
(a)
