# Supplementary material for: Novel age-associated DNA methylation changes and epigenetic age acceleration in middle-aged African Americans and whites
Source: Clin Epigenetics. 2019 Aug 19;11:119. doi: 10.1186/s13148-019-0722-1 (PMC6700815; doi:10.1186/s13148-019-0722-1)
Supplement: Supplementary file 3 — Figure S2. Scatter plots and Pearson’s correlation coefficients of methylation M values of top ten age-associated differentially methylated CpG positions (aDMPs) and chronological age in (a) African Americans and (b) whites. (PPTX 121 kb) [file 13148_2019_722_MOESM3_ESM.pptx]

## Slide 1
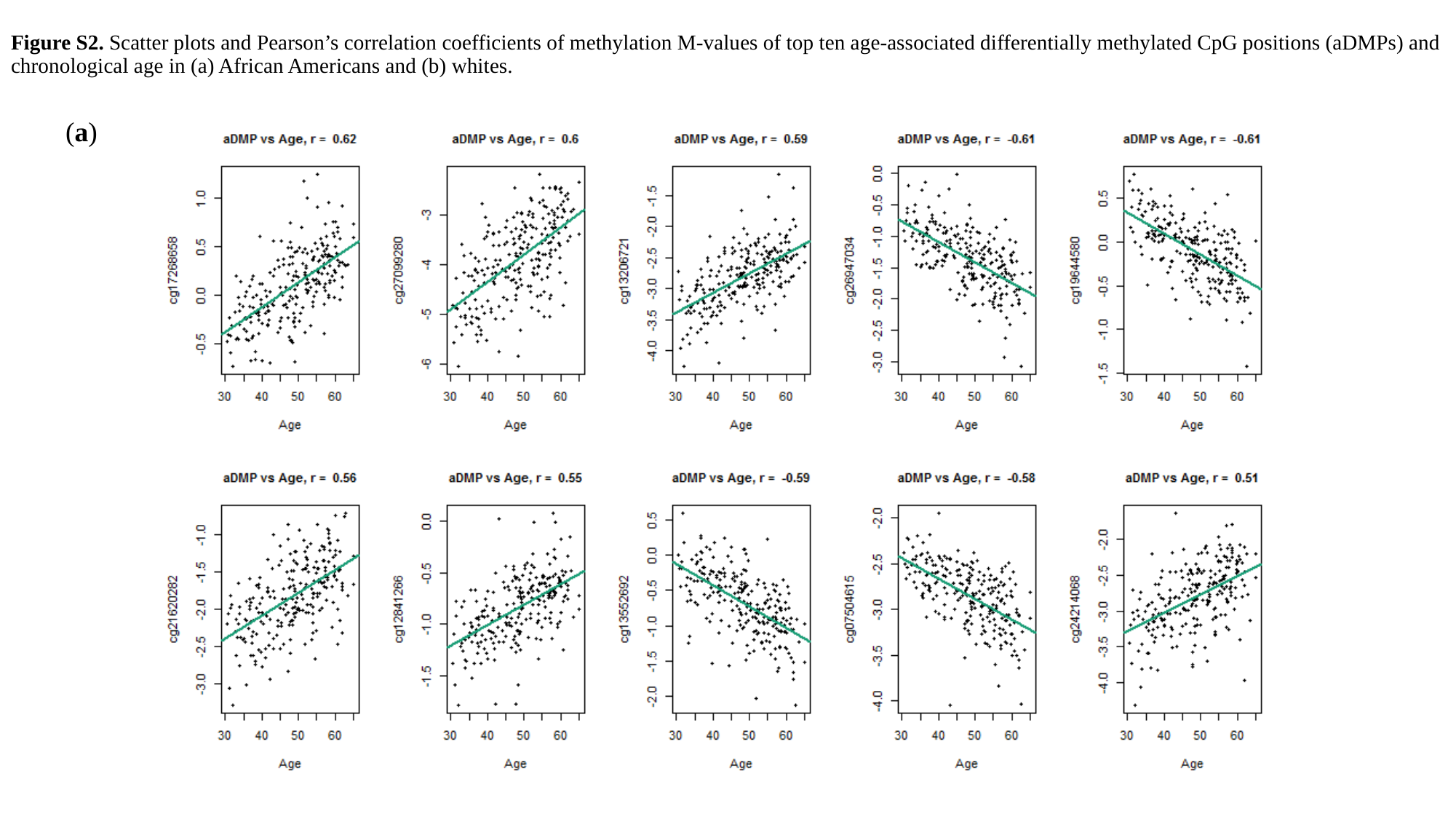

# Figure S2. Scatter plots and Pearson’s correlation coefficients of methylation M-values of top ten age-associated differentially methylated CpG positions (aDMPs) and chronological age in (a) African Americans and (b) whites.
(a)

## Slide 2
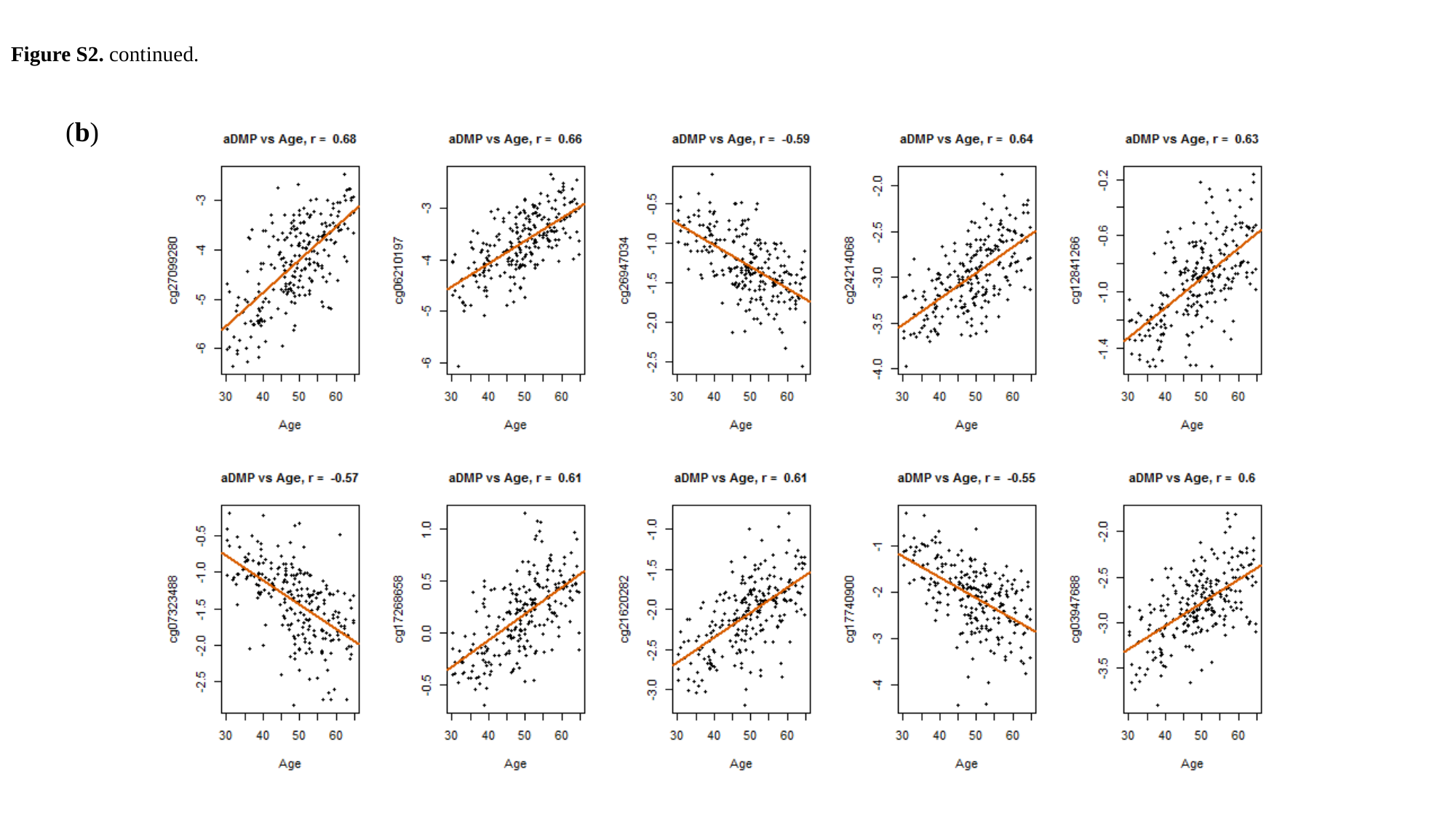

# Figure S2. continued.
(b)
