# Supplementary material for: Novel age-associated DNA methylation changes and epigenetic age acceleration in middle-aged African Americans and whites
Source: Clin Epigenetics. 2019 Aug 19;11:119. doi: 10.1186/s13148-019-0722-1 (PMC6700815; doi:10.1186/s13148-019-0722-1)
Supplement: Supplementary file 10 — Figure S3. Quantile-quantile plots of expected and observed p values of the association between DNA methylation and chronological age in African Americans (green) and whites (magenta) A) uncorrected and B) corrected using empirical null distribution. Genomic inflation measures are shown in parentheses. Histogram of test statistic expected (green) and observed (red) p values of the association between DNA methylation and chronological age in African Americans and whites C) uncorrected and D) corrected using empirical null distribution. Density plot of expected (black) and observed (red) p values of the association between DNA methylation and chronological age in African Americans and whites E) uncorrected and F) corrected using empirical null distribution. (PPTX 234 kb) [file 13148_2019_722_MOESM10_ESM.pptx]

## Slide 1
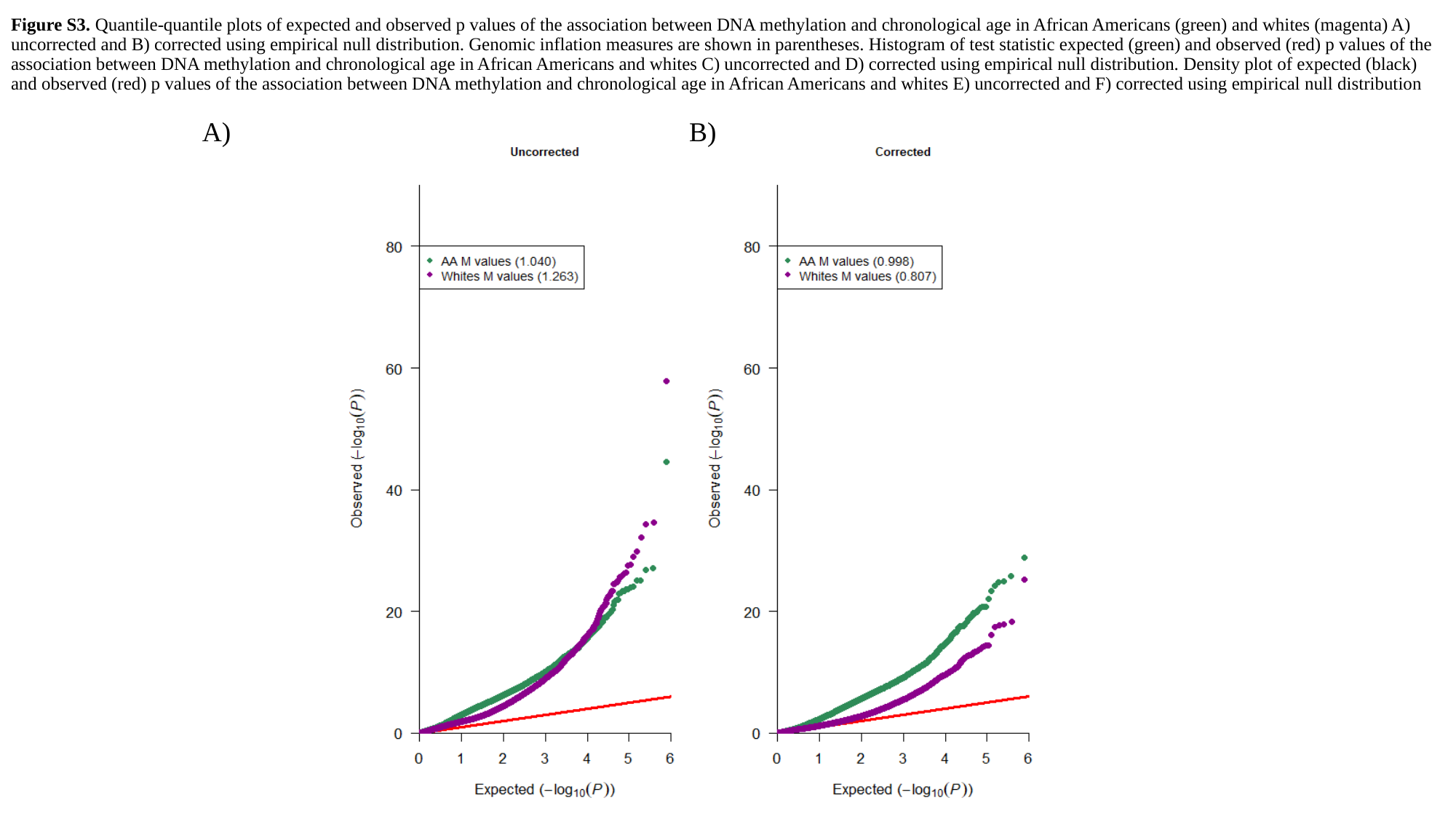

# Figure S3. Quantile-quantile plots of expected and observed p values of the association between DNA methylation and chronological age in African Americans (green) and whites (magenta) A) uncorrected and B) corrected using empirical null distribution. Genomic inflation measures are shown in parentheses. Histogram of test statistic expected (green) and observed (red) p values of the association between DNA methylation and chronological age in African Americans and whites C) uncorrected and D) corrected using empirical null distribution. Density plot of expected (black) and observed (red) p values of the association between DNA methylation and chronological age in African Americans and whites E) uncorrected and F) corrected using empirical null distribution
A)
B)

## Slide 2
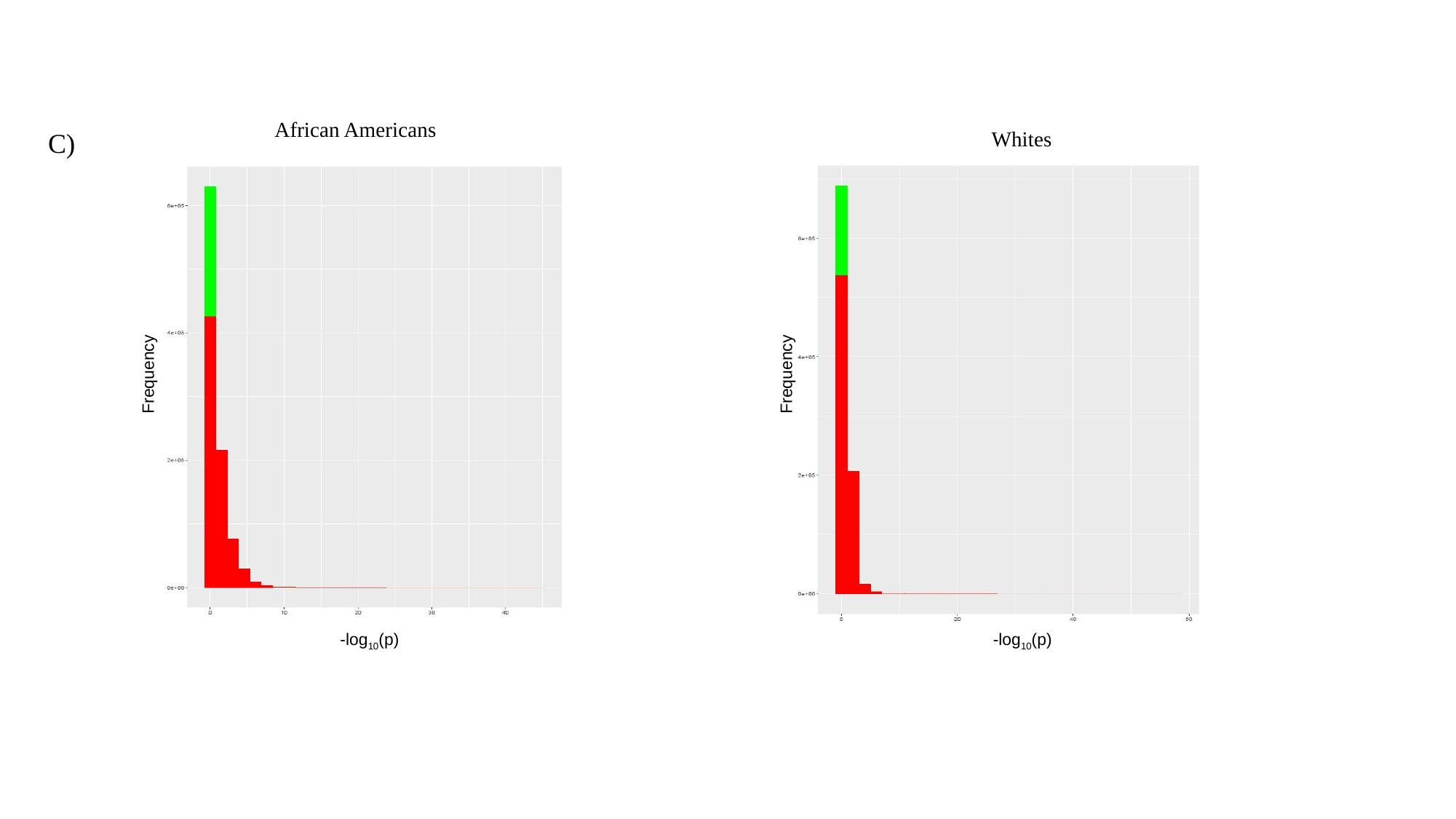

African Americans
Frequency
-log10(p)
Whites
Frequency
-log10(p)
C)

## Slide 3
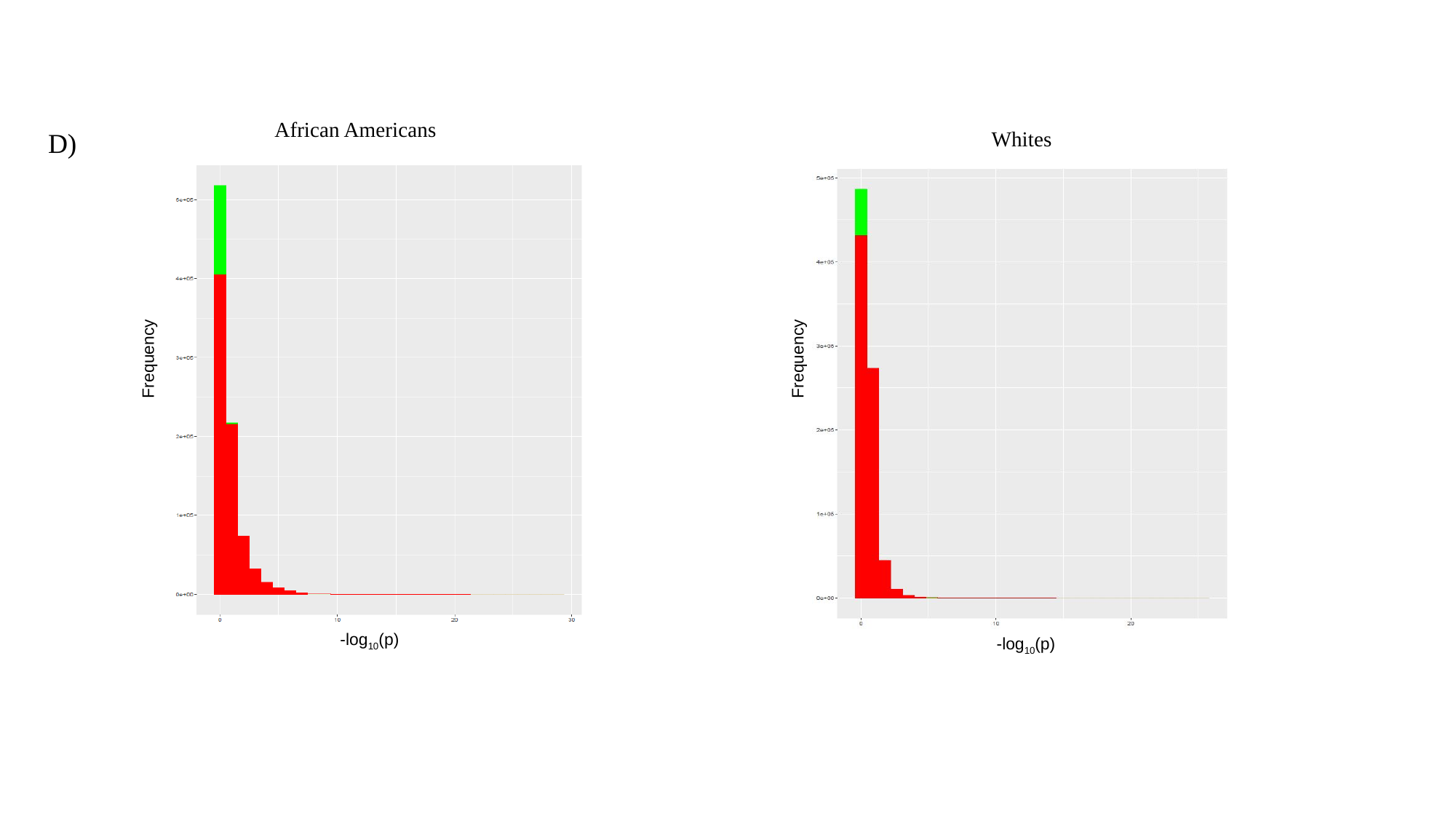

African Americans
Frequency
-log10(p)
Whites
Frequency
-log10(p)
D)

## Slide 4
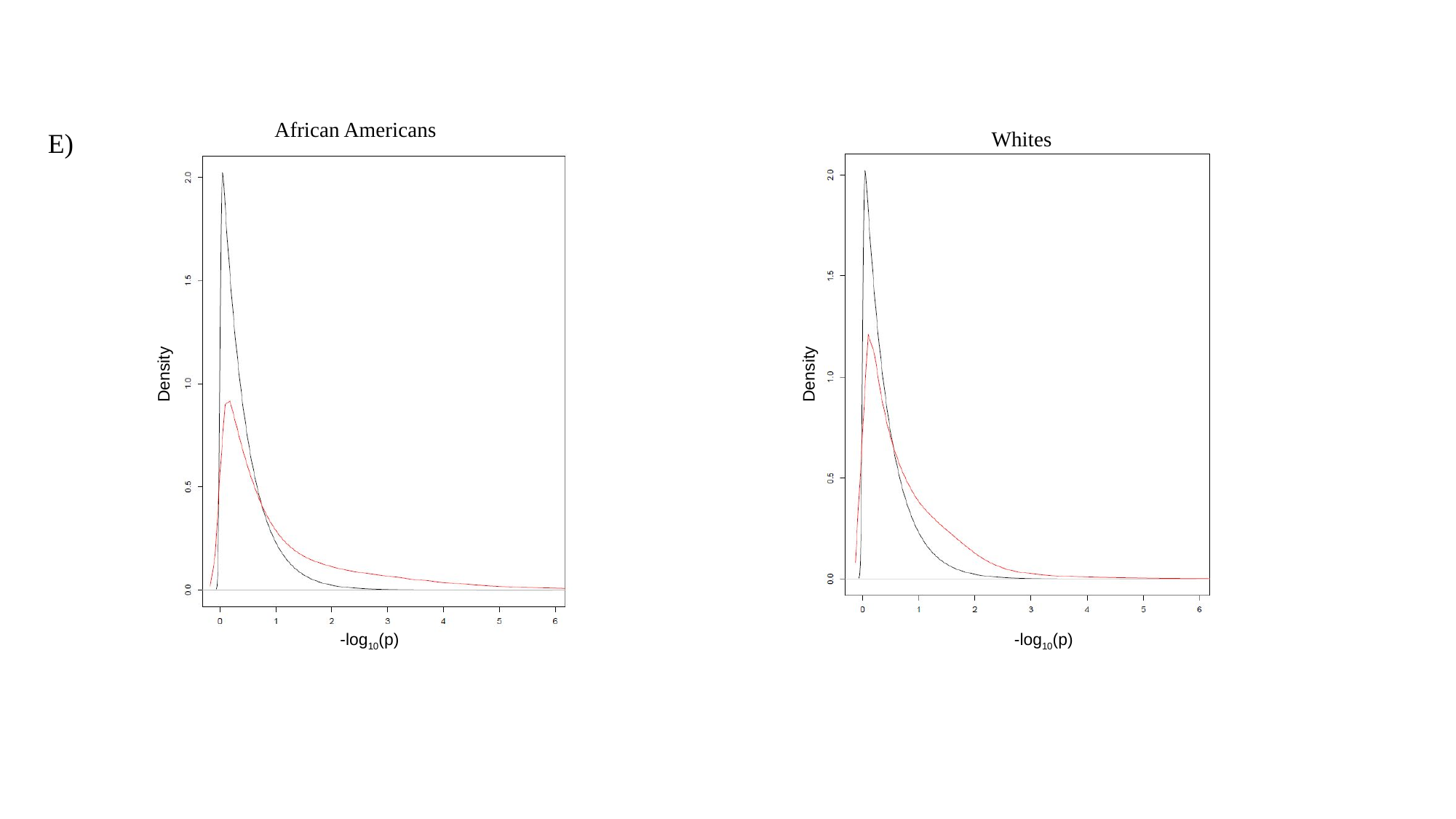

African Americans
Density
-log10(p)
Whites
Density
-log10(p)
E)

## Slide 5
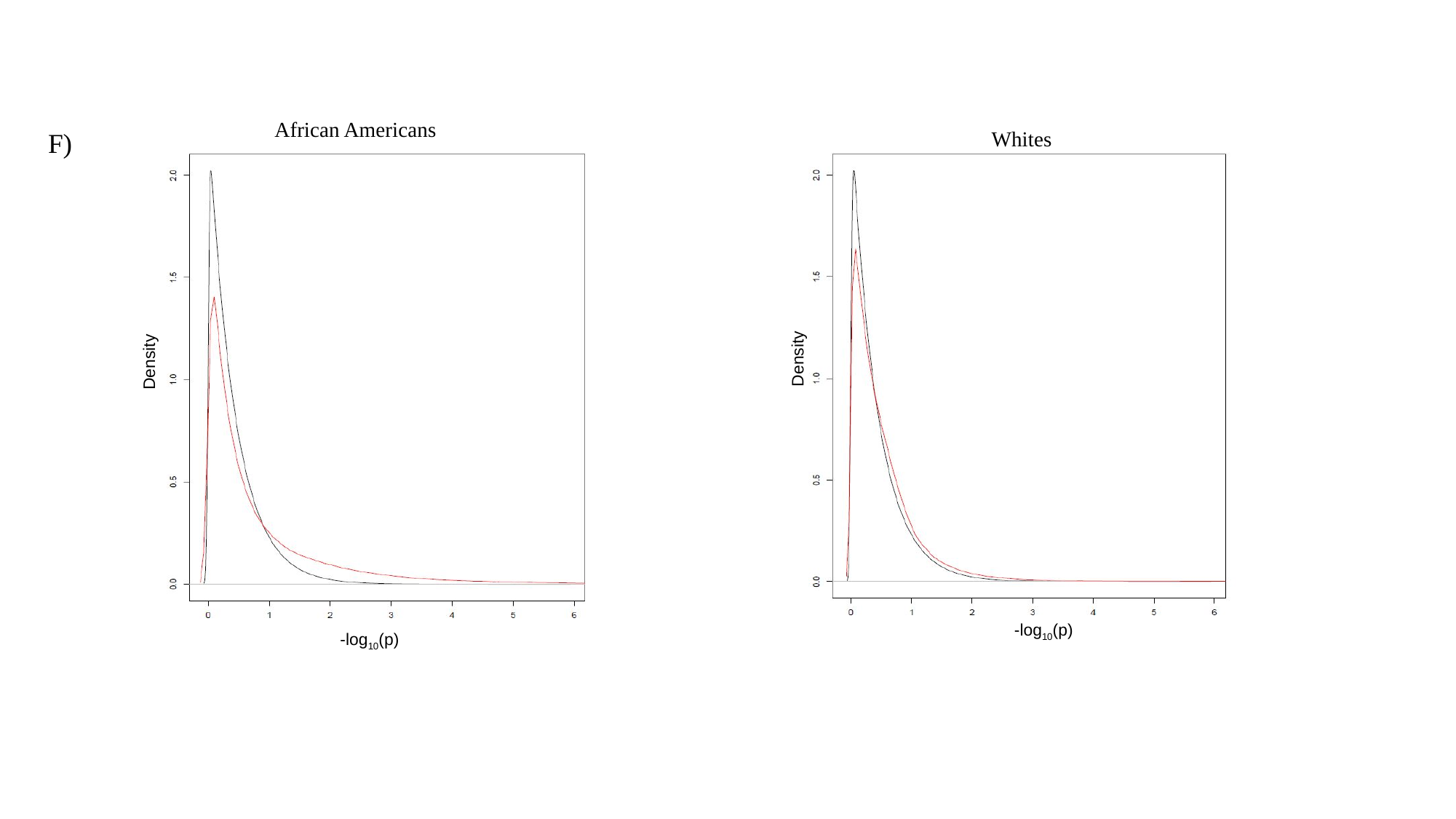

African Americans
Density
-log10(p)
Whites
Density
-log10(p)
F)
