# Supplementary material for: Novel age-associated DNA methylation changes and epigenetic age acceleration in middle-aged African Americans and whites
Source: Clin Epigenetics. 2019 Aug 19;11:119. doi: 10.1186/s13148-019-0722-1 (PMC6700815; doi:10.1186/s13148-019-0722-1)
Supplement: Supplementary file 11 — Figure S4. Volcano plots showing the distribution of age-associated differentially methylated CpG positions (aDMPs) with their effect size in M values and significance p value in the a) African American (AA) and b) white participants of the HANDLS study. (PPTX 197 kb) [file 13148_2019_722_MOESM11_ESM.pptx]

## Slide 1
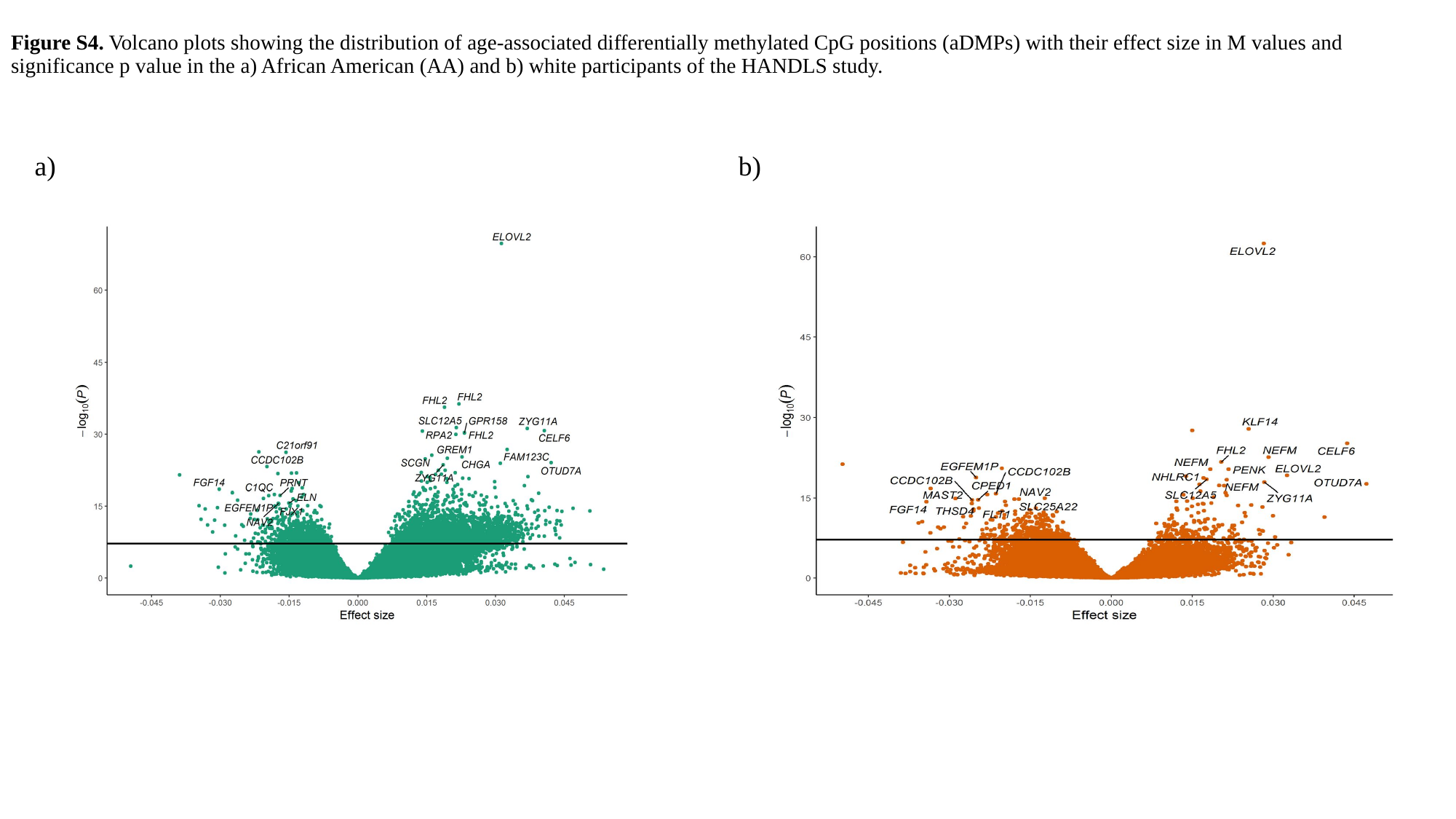

# Figure S4. Volcano plots showing the distribution of age-associated differentially methylated CpG positions (aDMPs) with their effect size in M values and significance p value in the a) African American (AA) and b) white participants of the HANDLS study.
a)
b)
